# The differential assimilation of nitrogen fertilizer compounds by soil microorganisms

**DOI:** 10.1093/femsle/fnae041

**Published:** 2024-06-07

**Authors:** Alice F Charteris, Timothy D J Knowles, Andrew Mead, Michaela K Reay, Katerina Michaelides, Richard P Evershed

**Affiliations:** Organic Geochemistry Unit, School of Chemistry, University of Bristol, Cantock’s Close, Bristol BS8 1TS, United Kingdom; Sustainable Agriculture Sciences, Rothamsted Research, North Wyke, Okehampton, Devon EX20 2SB, United Kingdom; Organic Geochemistry Unit, School of Chemistry, University of Bristol, Cantock’s Close, Bristol BS8 1TS, United Kingdom; Computational and Analytical Sciences, Rothamsted Research, Harpenden, Hertfordshire AL5 2JQ, United Kingdom; Organic Geochemistry Unit, School of Chemistry, University of Bristol, Cantock’s Close, Bristol BS8 1TS, United Kingdom; School of Geographical Sciences, University of Bristol, University Road, Bristol BS8 1SS, United Kingdom; Organic Geochemistry Unit, School of Chemistry, University of Bristol, Cantock’s Close, Bristol BS8 1TS, United Kingdom

**Keywords:** ^15^N stable isotope tracing (SIP), amino acids, nitrate, ammonium, urea, immobilization

## Abstract

The differential soil microbial assimilation of common nitrogen (N) fertilizer compounds into the soil organic N pool is revealed using novel compound-specific amino acid (AA) ^15^N-stable isotope probing. The incorporation of fertilizer ^15^N into individual AAs reflected the known biochemistry of N assimilation—e.g. ^15^N-labelled ammonium (^15^NH_4_^+^) was assimilated most quickly and to the greatest extent into glutamate. A maximum of 12.9% of applied ^15^NH_4_^+^, or 11.7% of ‘retained’ ^15^NH_4_^+^ (remaining in the soil) was assimilated into the total hydrolysable AA pool in the Rowden Moor soil. Incorporation was lowest in the Rowden Moor ^15^N-labelled nitrate (^15^NO_3_^−^) treatment, at 1.7% of applied ^15^N or 1.6% of retained ^15^N. Incorporation in the ^15^NH_4_^+^ and ^15^NO_3_^−^ treatments in the Winterbourne Abbas soil, and the ^15^N-urea treatment in both soils was between 4.4% and 6.5% of applied ^15^N or 5.2% and 6.4% of retained ^15^N. This represents a key step in greater comprehension of the microbially mediated transformations of fertilizer N to organic N and contributes to a more complete picture of soil N-cycling. The approach also mechanistically links theoretical/pure culture derived biochemical expectations and bulk level fertilizer immobilization studies, bridging these different scales of understanding.

## Teaser

Adapted from: Charteris AF. Overview and Future Work. In *^15^N Tracing of Microbial Assimilation, Partitioning and Transport of Fertilisers in Grassland Soils*. Springer Theses, Springer Nature, Switzerland AG, 2019, 239.

## Introduction

Nitrogen (N) fertilizers are essential to modern food production and 105 Tg N fertilizers were used in 2016 (FAO 2019). It is estimated, however, only 17% of N applied to crops ultimately supports human nutrition, with the remainder being lost to the environment during food production and processing (Leach et al. 2012, Fowler et al. 2013). This brings the low nutrient use efficiency of the human food-chain into critical focus. The interaction of applied fertilizer N with the soil N-cycle, and influence on soil organic N, represents an important determinant of the fate of fertilizer N, the N balance of soil and eventual efficiency of production systems. Major gaps exist regarding the biological processing of N fertilizers in soils, particularly the routes and proportions of conversion into soil organic N.

Processing of N fertilizers has traditionally been quantified using isotope pool dilution to determine rates of N mineralization, immobilization, and nitrification in soils. However, even in agricultural soils, N stored in organic forms dominates inorganic N (Dungait et al. 2012). This large and heterogeneous soil N pool still underpins soil N dynamics and the supply of N to microorganisms, plants, and loss pathways (in some cases providing 30%–50% of the inorganic N for crop uptake; Macdonald et al. 1997, Murphy et al. 2000, Dungait et al. 2012). In order to provide a new perspective on the biomolecular fate and partitioning of different common N fertilizer compounds into soil organic N, we herein describe the application of compound-specific amino acid (AA) ^15^N-stable isotope probing (SIP) to investigate N-cycling into the soil protein pool (Charteris et al. 2016). The approach combines compound-specific gas chromatography–combustion-isotope ratio mass spectrometry (GC–C-IRMS) and ^15^N-SIP in the meta-metabolome of the whole soil system (Knowles et al. 2010; or other complex media, e.g. river water; Mena-Rivera et al. 2022) and is essentially a targeted ^15^N fluxomics approach (Cascante and Marin 2008). The soil protein pool is the largest (20%–50% of total soil N), and arguably most important, identifiable class of soil organic N (Stevenson 1982). Microbially mediated N transformations through the AA glutamate (Glu; Santero et al. 2012) represent the gateway between the inorganic and organic soil N pools (Supplementary Fig. S1). The extent to which fertilizer N is incorporated into soil protein has implications for its temporal availability to plants and loss pathways [e.g. nitrate (NO_3_^−^) leaching, ammonia (NH_3_) volatilization, and nitrous oxide (N_2_O) emissions] and we can now reveal distinct differences between three different fertilizer N compounds in two different grassland soils.

## Materials and methods

We explore whether differences exist in the processing of three different 10 atom % ^15^N-labelled fertilizer N compounds—potassium nitrate (K^15^NO_3_), ammonium chloride (^15^NH_4_Cl), and urea (CO(^15^NH_2_)_2_); henceforth referred to as the ^15^NO_3_^−^ treatment; the ^15^NH_4_^+^ treatment, and the ^15^N-U treatment, respectively—in two different soils, identified by site—Rowden Moor (RM) and Winterbourne Abbas (WA)—using soil microcosms (Table 1).

**Table 1.** Table summarizing the laboratory incubation experiments conducted.

Soil	Substrate	Key	Labelling	Substrate applied 10^−1^ g soil	Mass N 10^−1^ g soil	Equivalent^a^ fertilization rate kg^−1^ N ha^−1^ year^−1^	Incubation periods
RM	K^15^NO_3_	RM-^15^NO_3_^−^	10 atom % ^15^N	400 µg in 200 µl DDW	55 µg	100	1.5, 3, 6, and 12 hours and 1, 2, 4, 8, 16, and 32 days
	^15^NH_4_Cl	RM-^15^NH_4_^+^	10 atom % ^15^N	400 µg in 200 µl DDW	105 µg	190	1.5, 3, 6, and 12 hours and 1, 2, 4, 8, 16, and 32 days
	CO(^15^NH_2_)_2_	RM-^15^N-U	10 atom % ^15^N	400 µg in 200 µl DDW	187 µg	340	3 hours and 2, 16, and 32 days
	Negative control	RM-C	―	200 µl DDW	―	―	0 hours and 32 days
WA	K^15^NO_3_	WA-^15^NO_3_^−^	10 atom % ^15^N	400 µg in 200 μl DDW	55 µg	100	1.5, 3, 6, and 12 hours and 1, 2, 4, 8, 16, and 32 days^b^
	^15^NH_4_Cl	WA-^15^NH_4_^+^	10 atom % ^15^N	400 µg in 200 μl DDW	105 µg	190	1.5, 3, 6, and 12 hours and 1, 2, 4, 8, 16, and 32 days^c^
	CO(^15^NH_2_)_2_	WA-^15^N-U	10 atom % ^15^N	400 µg in 200 μl DDW	187 µg	340	2 hours and 2, 16, and 32 days
	Negative control	WA-C	―	200 µl DDW	―	―	0 hours and 32 days

a Equivalent fertilization rate calculated based on a 0.3-m soil depth and an average of five to six treatments between February and October. The rates are generally within the range recommended for grasslands for dairy grazing (140–340 kg N ha^−1^ year^−1^; Defra 2010).

b Not all time-points analysed for AAs, only 3 and 6 hours and 2, 4, 16, and 32 days.

c Not all time-points analysed for AAs, only 1.5, 3, and 12 hours and 2, 8, and 32 days.

### Sites and soil sampling

Soil was sampled to a depth of 15 cm along a random W transect from plot six of RM experimental site at Rothamsted Research North Wyke, Devon, UK (50°46'42" N, 3°54'47" W) and from Little Broadheath field of Longlands Dairy Farm, near WA in Dorset, UK (50°42'46" N, 2°34'55" W). The RM soil is classified as a Stagni-vertic cambisol (FAO), a clayey noncalcareous Pelostagnogley of the Hallsworth series (British Classification), or a Typic haplaquept (USDA; Harrod and Hogan 2008). The Little Broadheath soil is a lime-rich clay loam of variable depth (0.3–0.8 m), underlain by chalk.

The RM site was a long-term grassland (>40 years) dominated by *Lolium* spp. interspersed with *Cynosurus, Festuca, Agrostis, Holcus*, and *Dactylis* spp. It had been grazed by cattle for around 25 years and had received ~200–250 kg N ha^−1^ year^−1^ as cattle slurry. The WA site, on the other hand, had been used for spring cropping before being converted to a grass ley (*Lolium perenne* and *Trifolium repens*) and used for dairying with a mobile milking parlour for 2 years prior to sampling. The ley was fertilized with 40 kg N ha^−1^ (previously as ammonium sulfate [(NH_4_)_2_SO_4_] and then as sulfur-coated urea [CH_4_N_2_O]) every 40 days from spring until the start of the ‘closed period’ on 15th September. which prohibits N fertilizer application on grasslands in nitrate vulnerable zones (Defra 2013). The samples of each soil were combined in equal weights and homogenized to produce a pooled soil sample for each site. Pooled samples were air-dried to allow sieving to <2 mm and then double distilled water (DDW) added to attain 50% water holding capacity (WHC).

### Incubations

Each experimental unit consisted of 10 g soil at 50% WHC contained in a 10-cm high by 2-cm diameter glass tube. Maintenance of the soil at 50% WHC was selected to prevent leaching and the tubes were fitted with furnaced and pierced aluminium foil lids to minimize volatile and evaporative losses. All incubations were carried out in triplicate so there were three tubes for each time point of each treatment. Incubation treatments and periods are summarized in Table 1. Treatments were injected into the soil and distributed over the full core depth. Incubations were halted at the required time by immersion in liquid nitrogen (N_2_) and stored at −20°C prior to freeze-drying. Whole freeze-dried soil cores were finely ground and homogenized using a pestle and mortar and stored in sealed 28 ml vials at −20°C.

### Extraction, isolation, and derivatization of hydrolysable AAs

Freeze-dried and ground incubation soil samples (100 mg) with an added internal standard of 100 µl norleucine in hydrochloric acid (400 µg ml^−1^ Nle in 0.1 M HCl) were hydrolyzed with 5 ml 6 M HCl at 100°C for 24 hours under an atmosphere of N_2_ (Fountoulakis and Lahm 1998, Roberts and Jones 2008). Acid hydrolysis extracts both free and proteinaceous AAs as well as catalyzing the breakdown of living microbial biomass (Roberts and Jones 2008). The relatively harsh conditions are necessary for the cleavage of peptide bonds between hydrophobic residues [e.g. isoleucine (Ile), leucine (Leu), and valine (Val)], but also result in the deamination of asparagine (Asn) to Asp and glutamine (Gln) to Glu and the complete destruction of cysteine (Cys) and tryptophan (Trp; Fountoulakis and Lahm 1998, Roberts and Jones 2008). The technique may also partially destroy serine (Ser; *ca*. 10% loss), threonine (Thr; *ca*. 5% loss), and tyrosine (Tyr; loss depends on level of trace impurities in hydrolysis agent; Fountoulakis and Lahm 1998) and has the potential to hydrolyse AA chains from nonproteinaceous sources, such as peptidoglycan, resulting in an overestimation of some AAs, mostly alanine (Ala), Glu, glycine (Gly), and lysine (Lys; Roberts and Jones 2008). The technique is, however, considered the most reliable method for determining the total protein content of soils (Roberts and Jones 2008) and as such, it is reasonable to equate total hydrolysable AA concentrations to the size of the soil protein pool. The hydrolysis is performed under N_2_ as the presence of O_2_ can induce the thermal breakdown of hydroxyl- and sulfur-containing AAs [e.g. Ser, Thr, Tyr, and methionine (Met); Roberts and Jones 2008]. Hydrolysates were collected by centrifugation, dried at 60°C under a stream of N_2_, and stored at −20°C under 1 ml 0.1 M HCl. Cation-exchange column chromatography with acidified Dowex 50WX8 200–400 mesh ion-exchange resin was used to isolate AAs from the hydrolysates (Metges and Petzke 1997). Finally, the hydrolysed soil AA mixtures were converted to their *N*-acetyl, *O*-isopropyl derivatives for analysis (Corr et al. 2007).

### Instrumental analyses

Bulk soil percentage total N (% TN) and *δ*^15^N analyses were carried out by elemental analysis-isotope ratio mass spectrometry (EA-IRMS) at the Lancaster node of the Natural Environment Research Council Life Sciences Mass Spectrometry Facility (NERC LSMSF). AAs as their *N*-acetyl, *O*-isopropyl derivatives were quantified by comparison with the Nle internal standard using gas chromatography–flame ionization detection (GC–FID). The *N*-acetyl, *O*-isopropyl AAs were identified by their known elution order and by comparison with *N*-acetyl, *O*-isopropyl derivatized-AA standards. Data were acquired and analysed using Clarity chromatographic station for Windows by DataApex. The *δ*^15^N values of individual AAs as their *N*-acetyl, *O*-isopropyl derivatives were determined using GC–C-IRMS. Data were acquired and analysed using Isodat NT 3.0 (Thermo Electron Corporation). Bulk soil percentage total C (% TC) analyses were carried out on a Eurovector EA3000 elemental analyser.

### Statistical information and calculations

AA plateau *Δ*^15^N values and % ^15^N_R_ incorporations were determined by curve fitting with a simple exponential equation using Genstat^®^ statistical software for biosciences (19th edition, VSNI):


(1) \begin{eqnarray*}
{y_i} = {\mathrm{\alpha }} + {\mathrm{\beta }}{{\mathrm{e}}^{ - {\mathrm{\theta }}{x_i}}} + {e_i},
\end{eqnarray*}

where *α* is the plateau AA *Δ*^15^N value or % ^15^N incorporation, *α* + *β* is the AA *Δ*^15^N value or % ^15^N incorporation at *t* = 0 (which is 0 by definition for these parameters) and *θ* is the rate at which AA *Δ*^15^N values or % ^15^N incorporations increase. In addition, due to the temporal trend of Glx *Δ*^15^N values in the ^15^NH_4_^+^ and ^15^N-U treatments, these responses were also fitted with a critical exponential regression:


(2) \begin{eqnarray*}
{y_i} = {\mathrm{\alpha }} + \left( {{\mathrm{\beta }} + {\mathrm{\gamma }}{x_i}} \right){{\mathrm{e}}^{ - {\mathrm{\theta }}{x_i}}} + {e_i},
\end{eqnarray*}

where *α* is again the plateau AA *Δ*^15^N value or % ^15^N incorporation and *α* + *β* is again the AA *Δ*^15^N value or % ^15^N incorporation at *t* = 0 (again 0 by definition).The balance between *γ* (increase) and *θ* (decay) controls the height and positioning (*x* value) of the peak in the critical exponential function, where *γ* can be used to assess the rate of increase in AA *Δ*^15^N values or % ^15^N incorporated (larger *γ* = faster, although comparison between *γ* values becomes less clear where *θ* values differ).

Lack of error bar overlap between mean *Δ*^15^N values at *t* = 32 days was used as an indicator of significant statistical difference between final AA *Δ*^15^N values. This approach was used because formal statistical testing would confirm a significant statistical difference between means with separated error bars, and would, rather, only be useful to determine whether there were any statistically significant differences between means with some error bar overlap. This further level of inspection was not deemed to add sufficient value to the interpretation of this work as the complex statistical modelling required to rigorously determine the statistical difference between plateau *Δ*^15^N values (using constrained curve fitting) would not be proportionate for the additional information obtained. Simple *t*-tests or analysis of variance using final *t* = 32-day values would be based on very small datasets and would therefore only provide confirmation where errors bars are separated, which can already be observed.

The percentage of the applied ^15^N incorporated into each AA is as follows:


(3) \begin{eqnarray*}
{\mathrm{\% \,\,}}{}_{}^{15}{{\mathrm{N}}_{\mathrm{A}}}{\mathrm{\,\,\textit{incorporation}}} = \left( {\frac{E}{{{n^{\mathrm{E}}}{{\left( {{}_{}^{15}{\mathrm{N}}} \right)}_{\mathrm{A}}}}}} \right) \times 100,
\end{eqnarray*}

where *E* is the ^15^N enrichment of the AA following application of a ^15^N-labelled substrate (taking into account the moles of N in the AA per gram of sample and the excess atom fraction of the AA after incubation, compared with the control). The percentage of retained ^15^N [based on *n*^E^(^15^ N)_P/C_, the excess moles of ^15^N present/retained per gram bulk sample at time, *t*] incorporated into each AA at time, *t* is as follows:


(4) \begin{eqnarray*}
{\mathrm{\% \,\,}}{}_{}^{15}{{\mathrm{N}}_{\mathrm{R}}}{\mathrm{\,\,\textit{incorporation}}} = \left( {\frac{E}{{{n^{\mathrm{E}}}{{\left( {{}_{}^{15}{\mathrm{N}}} \right)}_{{\mathrm{P}}/{\mathrm{C}}}}}}} \right) \times 100.
\end{eqnarray*}

Finally, the percentage of applied/retained ^15^N incorporated into newly synthesized soil protein was determined by summing the results of Equations (3) or (4), respectively, for individual AAs.

## Results and discussion

**Figure 1.**
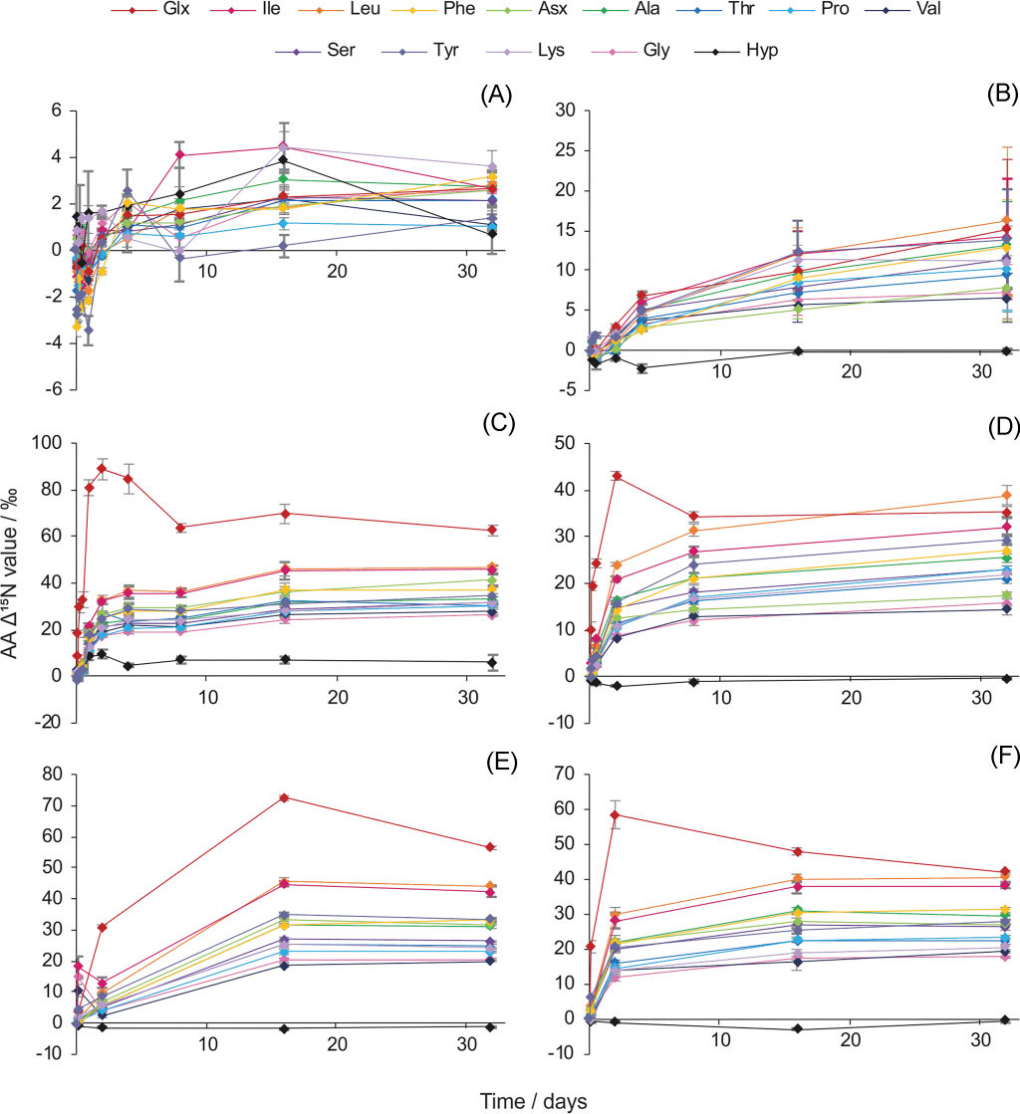
Time–course plots of AA *Δ*^15^N values revealing ^15^N assimilation into individual AAs in the six treatments. (A) RM-^15^NO_3_^−^ , (B) WA-^15^NO_3_^−^ (error bars at *t* = 16 and 32 days are coloured to aid differentiation), (C) RM-^15^NH_4_^+^, (D) WA-^15^NH_4_^+^, (E) RM-^15^N-U, and (F) WA-^15^N-U. RM and WA refer to the two different soils from the two sites, RM and WA and the three amendments were potassium nitrate (K^15^NO_3_), ammonium chloride (^15^NH_4_Cl), and urea (CO(^15^NH_2_)_2_). Error bars are ± SE (*n* = 3). See Supplementary Fig. S3. For individual figures for each AA in each treatment for additional clarity.

Ancillary data for the incubation experiments is given in Supplementary Note 1 and Supplementary Tables S1–S8. AA ^15^N-SIP exposes patterns in the biochemical assimilation pathways of applied ^15^N-labelled substrates via changes in the measured isotopic compositions (*δ*^15^N values) of each hydrolyzable AA over time (Charteris et al. 2016). AA *δ*^15^N values reflect the relative ^15^N content in the AA pool at that time, with any additional ^15^N (*cf. t* = 0 AA *δ*^15^N values, i.e. *Δ*^15^N values; Fig. 1A–F) being derived from the applied ^15^N-labelled substrate.

Individual AAs demonstrated different levels and patterns of ^15^N incorporation in each treatment, but in both ^15^NO_3_^−^ treatments, *Δ*^15^N values initially dipped before rising (Fig. 1A and B). All AAs exhibited a similar temporal pattern, but a range of responses (AA *Δ*^15^N values) was observed at all time points. In the ^15^NH_4_^+^ and ^15^N-U treatments (Fig. 1C–F), glutamate [abbreviated to ‘Glx’ since acid hydrolysis deaminates glutamine to glutamate, so the measured glutamate pool includes contributions from glutamic acid (Glu) and glutamine (Gln)] had a different trend from the two-phase rise of other AAs rising more quickly to an early peak (at *ca. t* = 2 days).

Explanations for the temporal trends in soil AA *δ*^15^N values following ^15^NO_3_^−^ and ^15^NH_4_^+^ treatments fit with the known biochemistry of N assimilation (Charteris et al. 2016, Supplementary Fig. S1). Ammonium is generally the preferred anabolic source of inorganic N for soil microorganisms as both NO_3_^−^ uptake and reduction to NH_4_^+^ for incorporation into cell material require more energy (and thus C; Rice and Tiedje 1989, Recous et al. 1990, Magasanik 1993, Geisseler et al. 2010). The suggestion of toxically high NO_3_^−^ concentrations inducing localized cell lysis (Charteris et al. 2016) may not adequately account for the early negative *Δ*^15^N values in the ^15^NO_3_^−^ treatments. Much of the material released from lysed cells would require mineralization prior to assimilation into AAs, which would take time (Kuzyakov et al. 2000). Instead, KNO_3_ could have stimulated the release of some clay fixed NH_4_^+^ (by replacement of NH_4_^+^ with K^+^; Nieder et al. 2011). Fast assimilation of this apparently ^15^N-depleted NH_4_^+^ (*cf*. other N sources for AA biosynthesis, perhaps due to some isotope effect(s) associated with NH_4_^+^ fixation and subsequent release) resulted in the biosynthesis of transiently ^15^N-depleted AAs. Temporal trends in AA *Δ*^15^N values in the ^15^N-U treatments are similar to those of the ^15^NH_4_^+^ treatments and urea-^15^N was most likely hydrolyzed (Mobley et al. 1995) and assimilated as ^15^NH_4_^+^ [via reductive amination of *α*-ketoglutarate to _L_-Glu catalyzed by glutamate dehydrogenase (GDH) or via the glutamine synthetase-glutamate synthase (GS-GOGAT) pathway; Supplementary Fig. S1; Santero et al. 2012]. The relative contribution of Glu and Gln to the Glx pool could not be determined in this study and may have influenced which ^15^NH_4_^+^ assimilation pathway dominated in the different soils (Geisseler et al. 2009). The relative operation of these pathways is also affected by other factors (e.g. the C:N ratio of the amendment; Geisseler et al. 2009). The contribution of Glu and Gln to the Glx pool can be expected to have been the same at the start of each incubation in the same soil receiving the different treatments.

Since AA concentrations (and thus the balance of AA degradation/biosynthesis/turnover) did not change markedly during the incubation experiments (Supplementary Note 1; Supplementary Tables S3–S8), ^15^N may be expected to be distributed (after initial uptake) in proportion to the quantity of N in each AA pool. However, ^15^N can only be incorporated into actively cycling pools, so a large, but stable AA pool would incorporate less ^15^N than expected based on the amount of N in that AA pool. Deviations from a proportional distribution, therefore, resulted from activity differences between AA pools and from the different biochemical routing of ^15^N. These deviations are reflected in differing fitted (Equations 1 and 2) or ‘plateau’ AA *Δ*^15^N values (if ^15^N is distributed in proportion with AA concentration, AA *Δ*^15^N values would be approximately equal for all AAs in a given experiment; Supplementary Note 2; Supplementary Tables S9 and S10).

AA *δ*^15^N (and *Δ*^15^N) values indicate the proportion of N derived from the applied ^15^N but not the total flux of that ^15^N into in each hydrolyzable AA [or, therefore, the distribution of applied ^15^N or ^15^N still present in the soil (*retained ^15^N*) amongst the AAs]. AAs present in higher concentrations require larger amounts of ^15^N to raise the N isotopic composition of the whole pool. It is, therefore, useful to consider the excess moles of ^15^N in each AA and, to provide some context, in comparison with the excess moles ^15^N applied (Equation 3), or alternatively, the excess moles ^15^N retained in the soil at that time (Equation 4; Supplementary Fig. S2). Percentage applied ^15^N incorporations (% ^15^N_A_ incorporation) are useful in providing an indication of the overall fate of applied ^15^N (affected by heterogenous treatment applications and any losses of ^15^N from the system, which would occur in a field). Percentage retained ^15^N incorporations (% ^15^N_R_ incorporation) reflect the partitioning of ^15^N present (or retained) in the system at the time, but as these data are calculated based on bulk soil *δ*^15^N values, could be affected by volatile losses of lighter ^14^N raising values.

**Figure 2.**
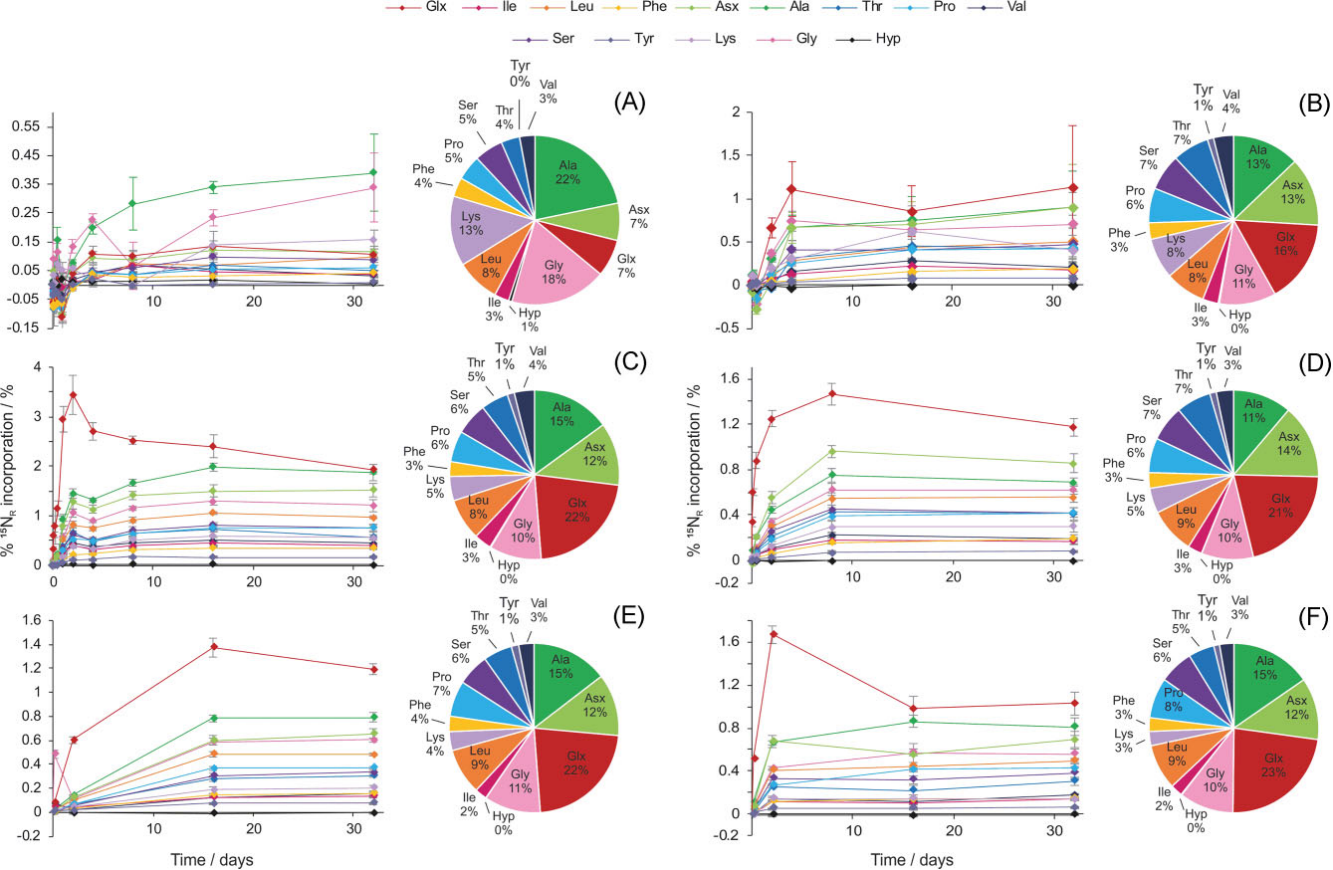
Time–course plots of AA % ^15^N_R_ incorporations revealing ^15^N assimilation into individual AAs in the six treatments, alongside pie charts of the relative percentage of retained ^15^N in each AA pool this represented, based on the plateau partitioning of ^15^N in each total hydrolyzable AA pool (derived from simple exponential regressions of the % ^15^N_R_ incorporated into AAs over time; Equation 1). (A) RM-^15^NO_3_^−^ (error bars for Ala and Gly are coloured to aid differentiation), (B) WA-^15^NO_3_^−^ (error bars for Glu, Asp, and Ala are coloured to aid differentiation), (C) RM-^15^NH_4_^+^, (D) WA-^15^NH_4_^+^, (E) RM-^15^N-U, and (F) WA-^15^N-U. Error bars are ± SE (*n* = 3). Adapted from Charteris (2019).

Temporal patterns in the % ^15^N_R_ incorporation into each AA under each treatment (Fig. 2A–F) were similar to those of increasing AA *Δ*^15^N values (Fig. 1A–F) but were dependent on the quantity of AA N in each pool (Supplementary Tables S3–S8; to reflect the routing/partitioning of ^15^N) and smoothed by the availability of ^15^N in the bulk soil. As for AA plateau *Δ*^15^N values, AA plateau % ^15^N_R_ incorporations were determined by fitting simple exponential regressions (as well as critical exponential regressions for Glx in the ^15^NH_4_^+^ and ^15^N-U treatments; Equations 1 and 2; Supplementary Tables S11 and S12). The largest plateau hydrolyzable AA % ^15^N_R_ incorporations were found in Glx in five out of the six treatments, ranging from 2.65 ± 0.15% of retained ^15^N in RM-^15^NH_4_^+^ to 1.0 ± 0.21% in WA-^15^NO_3_^−^ (Fig. 2A–F). Using an analogous experimental approach (kinetic flux profiling) on an *Escherichia coli* culture, Yuan et al. (2006) similarly found largest fluxes of ^15^N into Glu and Gln and surmized that Glu N was quickly transferred into other AAs (Reitzer 2003). The exception to this was the RM-^15^NO_3_^−^ treatment, in which the highest % ^15^N_R_ was observed in Ala (0.4% retained ^15^N). In general, and particularly in the ^15^NO_3_^−^ treatments, AAs present at higher concentrations (Supplementary Tables S3–S8) demonstrated larger % ^15^N_R_ incorporations (Fig. 2A–F), as might be expected to maintain the AA concentration profile of the soil, which did not vary. As highlighted by differences in AA *Δ*^15^N values, however, applied ^15^N was not homogeneously distributed across the AA pools due to differently responding subpools of AAs and/or the differential biochemical routing of ^15^N (Fig. 2A–F; Supplementary Note 3). That the plateau ^15^N levels (as depicted in the pie charts in Fig. 2C–F) for the ^15^NH_4_^+^ and ^15^N-U treatments are very similar, but those of the ^15^NO_3_^−^ are different both from these four and one another, suggests that the two soils responded differently to nitrate, but similarly to the other two substrates.

**Figure 3.**
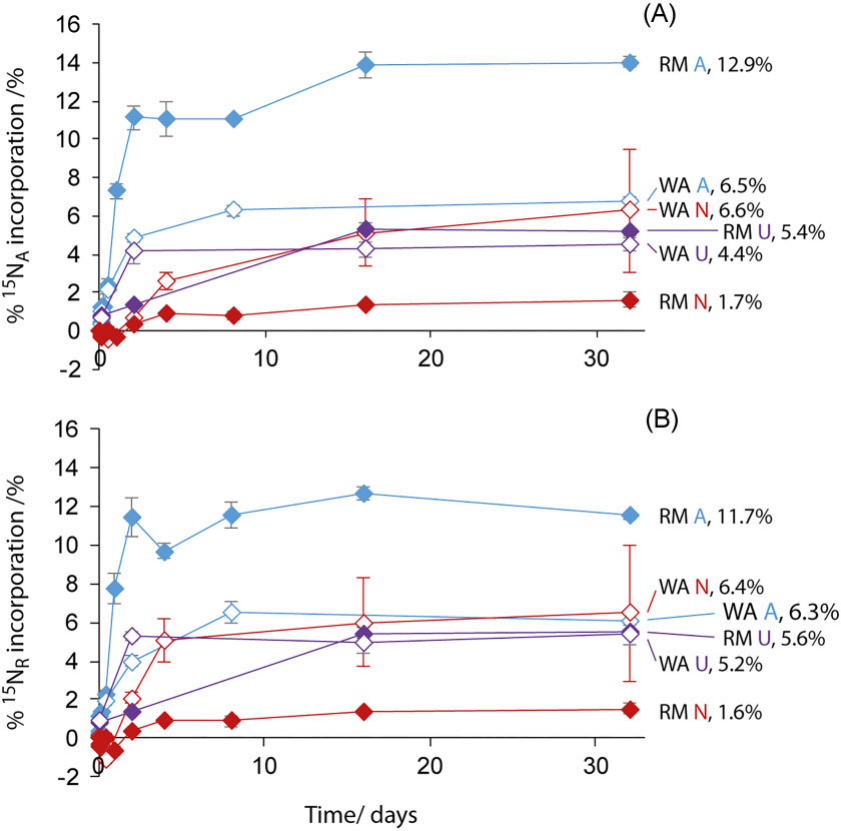
Percentage of ^15^N incorporated into the total hydrolyzable AA pool for all treatments, labelled with the plateau % ^15^N_R_ incorporations determined by simple exponential regressions. (A) Percentage of applied ^15^N and (B) Percentage of the ^15^N still present in the soil or ‘retained’ at that time. Error bars are ± SE (*n* = 3), the error bars of the WA-^15^NO_3_^−^ treatment are highlighted in red as the bar at *t* = 32 days is large and otherwise difficult to distinguish. Adapted from Charteris (2019).

A summation of the results of Equations (3) and (4) for each hydrolyzable AA gives the % ^15^N_A_ incorporation and % ^15^N_R_ incorporation into the total hydrolyzable AA pool, respectively (Fig. 3). There were only minor differences between the % ^15^N_A_ incorporation and % ^15^N_R_ incorporation into the total hydrolyzable AA pool, which were due to bulk soil ^15^N contents (Supplementary Table S1). As before, plateau % ^15^N incorporations into the total hydrolyzable AA pool were determined by fitting simple exponential regressions (Equation 1; Supplementary Table S16). Differences between the three N sources and two soils are clear—the three substrates are assimilated to significantly different extents (^15^NH_4_^+^ > ^15^N-U > ^15^NO_3_^−^) in the RM soil, but not in the WA soil (based on error bar overlap).

Although the two soils in these experiments were sampled from cattle-grazed grasslands in southwest England, they had different management histories and contrasting compositions (non-calcareous versus calcareous), which affected the biotic and abiotic processing of applied N (Müller et al. 2011). The RM soil received only cattle slurry for the 25 years prior to soil sampling while the WA soil also received regular additions of ammonium sulfate or urea (since 2011 when it was converted from spring crops to grass ley). Manuring, and higher soil percentage total organic carbon (% TOC) and percentage total N (% TN) contents have been related to greater soil microbial biomass activity (RM > WA; *t* = 0% TOC 6.80% *cf*. 4.17% and % TN 0.63% *cf*. 0.45; Söderström et al. 1983, Černý et al. 2003, Edmeades 2003, Booth et al. 2005, Müller et al. 2011).

Substrate assimilation in the RM soil matched expectations based on N assimilation biochemistry and previous studies assessing fertilizer N immobilization with bulk measurements (e.g. Wickramasinghe et al. 1985, Jackson et al. 1989, Recous et al. 1990, Christie and Wasson 2001). NO_3_^−^-^15^N was not used extensively as an anabolic N source. Both NO_3_^−^ uptake and incorporation into cell material (via reduction to NH_4_^+^) require more energy (and thus C) than NH_4_^+^ assimilation and NO_3_^−^ uptake can be inhibited by only low concentrations of NH_4_^+^ (Rice and Tiedje 1989, Recous et al. 1990, Magasanik 1993, Geisseler et al. 2010). Urea-^15^N incorporation was slower and less extensive than ^15^NH_4_^+^ incorporation as urea must first be hydrolyzed. Urease is ubiquitous in soils, however, and urea hydrolysis can occur extra- or intracellularly (Mobley et al. 1995, Geisseler et al. 2010), at a lower metabolic cost than ^15^NO_3_^−^ reduction.

The operation of a more active (or larger) soil microbial biomass in the RM soil is supported by the significantly higher (almost double) plateau level of incorporation of ^15^NH_4_^+^ (bioavailable N source) in this soil, compared with the WA soil. Alternatively, less of the applied ^15^NH_4_^+^ may have become unavailable (e.g. by organic matter adsorption or clay-fixation; Booth et al. 2005, Nieder et al. 2011) in the RM soil. Lower NH_4_^+^ availability in the WA soil, compared with the RM soil could also explain the significantly greater assimilation of the less favourable N source, NO_3_^−^(-^15^N), by the WA soil, (where not limited by C). In addition, the WA soil may have become better adapted to NO_3_^−^-anabolism due to historic inorganic fertilization (Inselsbacher et al. 2010, Bunch and Bernot 2012), which can result in soil NO_3_^−^ accumulation from nitrification due to NH_4_^+^ assimilation saturation or out-competition under C-limitation (Robertson and Groffman 2007). Indeed, attunement to urea fertilization of this soil could also be responsible for the faster (initial and overall) assimilation of urea-^15^N compared with the RM soil through increased endogenous urease concentrations.

Further, differences in the active microbial community, such as relative bacterial and fungal ratios, arising from differing management, may also influence dynamics of uptake for differing N amendments. Other work at the RM site using amino sugar (AS) ^15^N-SIP allowed quantification of ^15^N assimilation in this smaller, but more specific soil organic N pool (Reay et al. 2019a, Joergensen 2018). Assimilation into bacterial AS pools reflected dynamics observed herein for AAs (Reay et al. 2019b), while fungal AS exhibited slower uptake, and a lower preference for NH_4_^+^ over NO_3_^−^, likely reflecting uptake of secondary N sources (Marzluf 1997, He et al. 2011). Hence the differing soil types, and management at the RM and WA sites herein likely resulted in differing microbial communities (Malik et al. 2018, Romdhane et al. 2022), and thus attunement to N amendments.

Overall, a maximum of 12.9% of applied ^15^N (as ^15^NH_4_^+^), or 11.7% of ‘retained’ ^15^N was assimilated into the total hydrolyzable AA pool (in RM-^15^NH_4_^+^; Fig. 3; Supplementary Table S16). Incorporation was lowest in RM-^15^NO_3_^−^, at 1.7% of applied ^15^N, or 1.6% of retained ^15^N. These maximal plateau % ^15^N incorporations are unlikely to have been caused by ^15^N-substrate limitation during the incubations since ^15^N remained in the soil (based on bulk soil *δ*^15^N values) and other processes: are either considered poor competitors for NH_4_^+^ (e.g. nitrification); would not reduce ^15^N availability (e.g. denitrification or other gaseous losses, which were not observed to occur extensively, and would likely increase, rather than decrease, bulk soil *δ*^15^N values); or were not observed to occur (e.g. ^15^N loss via leaching). Maintenance of the soil at 50% WHC prevented leaching losses and made anaerobic microsites suitable for denitrification and dissimilatory nitrate reduction to ammonium (DNRA) less likely to develop (Tiedje et al. 1984, Sexstone et al. 1985). Rather, maximal ^15^N assimilations probably resulted from regulation of N uptake/assimilation as limitation by another essential nutrient (e.g. C or P) arose in the soil. Physical and chemical protection of soil organic C reduces microbial availability, resulting in C-limitation, which is consistent with lower NO_3_^−^ assimilation observed in the WA soil, which had lower C content compared to the RM soil (Soong et al. 2019).

The application of our new ^15^N-AA SIP approach provides new insights into inorganic and organic N assimilation biochemistry by soil microbes. Critically, it provides vital mechanistic links between theoretical/pure culture derived biochemical expectations and bulk level fertilizer immobilization studies, bridging these different scales of understanding. Moreover, the work demonstrates that simple biochemical processes (N assimilation in this case) operating in physiologically relevant complex matrices are subject to additional biotic and abiotic environmental influences. This includes substrate supply by similarly influenced upstream processes and can overall result in quite different apparent process efficiencies in different settings (here, soils). Hence, the work constitutes a key step toward greater appreciation of the microbially mediated transformations of fertilizer N to organic N and contributes to a more complete picture of soil N-cycling in response to fertilizer N applications. Finally, the quantitative estimates regarding these transformations generated through time–course incubation experiments are vital parameters for the next generation of soil N-cycling models.

## Supplementary Material

fnae041_Supplemental_Files

## Data Availability

All relevant data are available in this article, its supplementary information files or at DOI (fnae041), which includes the full raw data for all figures.
