# Supplementary material for: The differential assimilation of nitrogen fertilizer compounds by soil microorganisms
Source: FEMS Microbiol Lett. 2024 Jun 7;371:fnae041. doi: 10.1093/femsle/fnae041 (PMC11223579; doi:10.1093/femsle/fnae041)
Supplement: fnae041_Supplemental_Files [file fnae041_supplemental_files.zip › Supplementary Information_FEMS_Revised_woTC.docx]

**Supplementary Information**

*Supplementary Note 1 – Incubation experiment ancillary data*

Nitrogen can exist in a wide range of oxidation states (-III to +V) and is applied as fertiliser in several forms. Urea is the most commonly used N fertiliser compound worldwide (FAOSTAT, <http://www.fao.org/faostat/en/#data/RFB>; accessed 05/02/2018), while various ammonium (NH_4_^+^) and nitrate (NO_3_^-^) salts and anhydrous ammonia (NH_3_) comprise the remainder of commonly used synthetic N fertilisers. Nitrate and NH_4_^+^ are low molecular weight inorganic N compounds with distinctly different chemical properties, bearing opposite charges and between them, spanning the range of possible N oxidation states (+V and -III, respectively). They play important, complementary roles in the soil N cycle and may be interconverted but are commonly subject to contrasting fates. Urea, on the other hand, is a low molecular weight organic N molecule. Its popularity results from its high stability, lower cost and high N content (46% by weight; Glibert *et al.*, 2006).

The application of a ^15^N-labelled fertiliser compound to the soil microcosms immediately raised bulk soil *δ*^15^N values from *t*=0 values of *ca.* 4.5 – 6.4‰ (Supplementary Table 1). The percentage of the applied ^15^N retained in the bulk soil is:

| $\% {}^{15}Nretention=\left( \frac{n^{E}{({}^{15}N)}_{P/C}}{n^{E}{({}^{15}N)}_{A}} \right)\times100$ | **Supplementary Equation 1** |
| --- | --- |

where *n*^E^(^15^N)_P/C_ is the excess moles of ^15^N present/retained per gram in a bulk enriched sample, compared with control samples and *n*^E^(^15^N)_A_ is the number of moles of excess ^15^N applied per gram sample (above natural abundance values for the substrate). Bulk soil *δ*^15^N values and the percentages of applied ^15^N these values represent (% ^15^N retention; Supplementary Equation 1) in individual microcosms varied due to differences in ^15^N application efficiency and subsequent soil processes (e.g. possibly greater volatile losses of ^14^N), but there were no clear trends over time within each treatment.

Disparities in mean bulk soil *δ*^15^N values between treatments (i.e. those of ^15^NO_3_^-^ < ^15^NH_4_^+^ < ^15^N-U treatments; Supplementary Table 1) were due to different N application rates (Table 1). Mean % ^15^N retentions, however, were generally close to 100% with small standard errors (SEs) in all treatments, except WA-^15^NO_3_^-^ (57 ± 5%). Here, mean % ^15^N retentions were reduced by low percentages at earlier time points (*t*=1.5 hours to 2 days mean: 38%; SE: 4.2%; n=18), but ^15^N retentions at later time points were close to 100% (*t*=8 to 32 days mean: 94%; SE: 2.7%; n=9). This was likely due to poor treatment application in the earlier microcosms, but greater volatile losses of lighter ^14^N from the soil with time (most likely via denitrification with possibly some contribution from dissimilatory nitrate reduction from ammonia [DNRA]; Giles *et al.*, 2012), leading to an increase in the ^15^N/^14^N ratio, could have contributed to this trend. At some time-points % ^15^N retentions were slightly over 100%, possibly due to minor changes in the mass of soil to which the treatments were applied due to moisture changes in the soil being weighed during the incubation set-up, or minor differences in treatment application. This can result in apparently more ^15^N being available in the soil than expected based on what was known to be applied.

Bulk soil percentage total carbon and nitrogen contents (% TC and % TN) and AA concentrations were not affected by the treatments (Supplementary Tables 2-8) indicating that the overall N status of the soil had not been perturbed.

*Supplementary Note 2 – Statistical difference between amino acid Δ^15^N values*

Amino acid plateau *Δ*^15^N values were determined by fitting a simple exponential regression (Equation 1) to the time-course data for each AA in every treatment (Supplementary Tables 9 and 10). While generally similar to mean *Δ*^15^N values at *t*=32 days, where the simple exponential regression was a good fit for the data, plateau *Δ*^15^N values provide an estimate of eventual *Δ*^15^N values taking into account the trend in all the data and may therefore be considered a more reliable estimate of the overall final *Δ*^15^N value for each AA as a result of the ^15^N treatment. In the RM-^15^NO_3_^-^ treatment, AA plateau *Δ*^15^N values ranged from 0.1 ± 1.3 (Tyr) to 4.8 ± 7.1‰ (Phe; Supplementary Table 10), with a large degree of overlap between error bars at *t*=32 days (Fig. 1a). ^15^N was distributed largely in proportion to the moles of N in each AA pool, likely as a result of low overall ^15^N-enrichments. Amino acid plateau *Δ*^15^N values ranged from *ca.* ‑1.1 (Hyp) to 19.5 ± 10.0‰ (Leu) in the WA-^15^NO_3_^-^ treatment (Supplementary Table 10) and only Hyp in the WA-^15^NO_3_^-^ treatment had a different (lower) mean *Δ*^15^N value at *t*=32 days (Fig. 1b). Plateau *Δ*^15^N values were similarly lowest for Hyp in the ^15^NH_4_^+^ and ^15^N-U treatments for both soils (Supplementary Table 10). The lack of ^15^N assimilation into Hyp in all treatments (*Δ*^15^N values are generally zero or negative throughout) was likely because it can only be biosynthesised from proline (Pro; due to its secondary amino structure, via a specialised route that requires peptide-bound Pro residues as a substrate; Adams, 1970; Adams and Frank, 1980). In addition, bacteria are not known to utilise Hyp in proteins or cell structural components, but only in bacterial antibiotics (e.g. etamycin and actinomycin) produced by specialist organisms.

Glutamate plateau *Δ*^15^N values were the highest (up to 75.1 ± 3.1‰ for the RM-^15^NH_4_^+^ treatment). There was less overlap in the *t*=32-day *Δ*^15^N values/plateau *Δ*^15^N values of AAs in the ^15^NH_4_^+^ and ^15^N-U treatments than for ^15^NO_3_^-^, with smaller overlapped groups (Figs. 1c-f; Supplementary Table 9). The hierarchy/order in *t*=32-day *Δ*^15^N values/plateau *Δ*^15^N values of AAs/groups was also similar in all four treatments. Higher plateau *Δ*^15^N values for the Glx pool in the ^15^NH_4_^+^ and ^15^N-U treatments resulted from preferential routing of the ^15^N through this AA, in accordance with the known biochemistry of N assimilation (Glu; Meers *et al.*, 1970; Tempest *et al.*, 1970; Miller and Stadtman, 1972; Santero *et al.*, 2012). Interestingly, the hierarchy in *t*=32-day *Δ*^15^N values of AAs/groups was similar in all four of these treatments (Figs. 1c-f). Leucine (Leu), for example, consistently had a high *t*=32-day *Δ*^15^N value suggesting there may have been some preferential routing of the ^15^N into the Leu pool, and/or this AA had a smaller less active/inactive sub-pool than other AAs.

It should also be noted, however, that any small differences in bulk soil *δ*^15^N or *Δ*^15^N values (which, having started from identical *t*=0 *δ*^15^N values and percentage total nitrogen contents [% TN] contents for a given soil, reflect the amount of ^15^N still present [or *retained*] in the soil at the time) between microcosms could affect measured AA *δ*^15^N values (i.e. if more/less ^15^N is present in the soil for incorporation into AAs), but this is not accounted for in AA *δ*^15^N or *Δ*^15^N values.

Due to the temporal trend of Glx *Δ*^15^N values in the ^15^NH_4_^+^ and ^15^N-U treatments, these data were secondarily fitted with a critical exponential regression (Equation 2), which improved the percentage of variance accounted for by the fitted equation (Supplementary Tables 9 and 10). In all four cases, this regression also gave lower plateau Glx *Δ*^15^N values, which were closer to *t*=32-day *Δ*^15^N values for all but the RM-^15^N-U treatment (mean Glx *t*=32-day *Δ*^15^N value of 56.5‰), for which the simple exponential regression determined a higher plateau *Δ*^15^N value (64.5‰), while the critical exponential regression determined a lower value (48.3‰). Use of the simple exponential regression parameters allows fairer comparison with other AAs, but the parameters derived from the critical exponential regression are more accurate for Glx.

*Supplementary Note 3 – Distribution of ^15^N between hydrolysable amino acids and rates of partitioning*

Commonalities in the plateau distribution of retained ^15^N in the total hydrolysable AA pool between AAs in each treatment are highlighted by the pie charts shown in Figure 2a-f. Hydrolysable AAs, other than Glx, with larger plateau proportions of ^15^N in all treatments were alanine (Ala), aspartate (abbreviated to ‘Asx’ since acid hydrolysis deaminates asparagine to aspartate, so the measured aspartate pool includes contributions from aspartic acid and asparagine) and glycine (Gly). Phenylalanine (Phe), tyrosine (Tyr) and valine (Val) commonly had low proportions of ^15^N, while Hyp contained the least ^15^N in all treatments except RM-^15^NO_3_^-^. For the RM-^15^NO_3_^-^ treatment, Tyr had the lowest % ^15^N_R_ incorporation (0.00 ± 0.008%; Supplementary Table 12). The distributions of plateau ^15^N levels for AAs in the ^15^NH_4_^+^ and ^15^N-U treatments in both soils (as depicted in the pie charts in Fig. 2c-f) were very similar, but those of the ^15^NO_3_^-^ treatment were different both between each soil and from the ^15^NH_4_^+^ and ^15^N-U treatments in both soils.

The rates at which ^15^N was partitioned into each AA can also be used to provide valuable information. Zero-order rate constants were determined using a linear regression over the (sometimes very) approximately linear early time-course % ^15^N_R_ incorporation data (first 4, 0.5 and 2 days for the ^15^NO_3_^-^, ^15^NH_4_^+^ and ^15^N-U treatments, respectively):

| $y_{i}=\theta_{0}x_{i}+ e_{i}$ | **Supplementary Equation 2** |
| --- | --- |

where θ_0_ is the initial zero order rate constant (Supplementary Tables 13 and 14). Zero-order rate constants over the initial linear phase of incorporation give the speeds at which ^15^N can be transferred from substrates to AAs, providing a measure of ‘biosynthetic proximity’ (Knowles *et al.*, 2010). In agreement with this, in all but the RM-^15^NO_3_^-^ treatment, zero order rate constants were highest for Glx, while those of Hyp (biosynthesised via complex route/commonly not produced) were consistently low. For the RM-^15^NO_3_^-^ treatment, Gly had the highest zero order rate constant (0.056 ± 0.012% day^-1^; Supplementary Table 14). Zero order rate constants generally reflected biosynthetic routes; Ile, for example, which is biosynthesised via Thr, had lower zero order rate constants than Thr in all experiments, as did Thr compared with Asx, from which the former is biosynthesised. In addition, AAs with more complex/multi-step processes such as the aromatic Phe and Tyr had consistently low zero order rate constants. Biosynthesis requires first the condensation of phosphoenolpyruvate (glycolytic intermediate) and erythrose 4-phosphate (pentose phosphate pathway intermediate) and then reaction via the intermediates shikimate, chorismate and prephenate to produce the required *α*-ketoacid skeletons for transamination to Phe or Tyr (Nelson and Cox, 2013). Good correlations between initial rates of biosynthesis and plateau % ^15^N_R_ incorporations were also found in all treatments indicating fluxes of ^15^N were greatest into AAs that could be biosynthesised via the shortest/easiest routes.

First order rate constants (obtained from simple exponential regressions), on the other hand, impart the overall rates at which AAs were biosynthesised from applied ^15^N (Supplementary Table 15). First order rates were more comparable between AAs within each experiment, but those of Hyp were consistently low. Rates of AAs in the RM-^15^NO_3_^-^, RM-^15^U and WA-^15^NO_3_^-^ treatments were generally higher than those in the RM-^15^NH_4_^+^ and WA-^15^NH_4_^+^ treatments, while the WA-^15^U treatment commonly had the lowest rates for a given AA.

References for Supplementary Information

Adams E. Metabolism of Proline and Hydroxyproline. In: Hall DA, Jackson DS (eds.). *International Review of Connective Tissue Research*. New York: Academic Press, 1970, 2-82.

Adams E, Frank L. Metabolism of proline and the hydroxyprolines. *Annu Rev Biochem* 1980;**49**:1005-61.

Charteris AF. Biosynthetic Routing, Rates and Extents of Microbial Fertiliser Nitrogen Assimilation in Two Grassland Soils. In *^15^N Tracing of Microbial Assimilation, Partitioning and Transport of Fertilisers in Grassland Soils*. Springer Theses, Springer Nature Switzerland AG, 2019, 79-151.

FAOSTAT. <http://www.fao.org/faostat/en/#data/RFB>. (5 February 2018, date last accessed).

Giles M, Morley N, Baggs EM, *et al*. Soil nitrate reducing processes: Drivers, mechanisms for spatial variation, and significance for nitrous oxide production. *Front Microbiol* 2012;**3**, DOI:10.3389/fmicb.2012.00407.

Glibert PM, Harrison J, Heil C, *et al.* Escalating worldwide use of urea: A global change contributing to coastal eutrophication. *Biogeochemistry* 2006;**77**:441-63.

Knowles TDJ, Chadwick DR, Bol R, *et al.* Tracing the rate and extent of N and C flow from ^13^C,^15^N-glycine and glutamate into individual de novo synthesised soil amino acids. *Org Geochem* 2010;**41**:1259-68, DOI: 10.1016/j.orggeochem.2010.09.003.

Meers JL, Tempest DW, Brown CM. ‘Glutamine(amide): 2-Oxoglutarate amino transferase oxido-reductase (NADP)’, an enzyme involved in the synthesis of glutamate by some bacteria. *J Gen Microbiol* 1970;**64**:178-94.

Miller RE, Stadtman ER. Glutamate synthase from *Escherichia coli*: An iron-sulfide flavoprotein. *J Biol Chem* 1972;**247**:7407-19.

Nelson DL, Cox MM. Biosynthesis of amino acids, nucleotides, and related molecules. In: Nelson DL, Cox MM (eds.). *Lehninger Principles of Biochemistry*, sixth ed. Basingstoke: Macmillan Higher Education, 2013, 881-928.

Santero E, Hervás A, Canosa I, *et al.* Glutamate Dehydrogenases: Enzymology, Physiological Role and Biotechnological Relevance. In: Canuto RA (ed.) *Dehydrogenases*. Published online: InTech, 2014, 289-91.

Tempest DW, Meers JL, Brown CM. Synthesis of glutamate in *Aerobacter aerogenes* by a hitherto unknown route. *Biochem J* 1970;**117**:405-7.

Supplementary figure legends

**Supplementary Figure 1. Nitrogen assimilation pathways, including nitrate reduction and urea hydrolysis. a**, The glutamate dehydrogenase (GDH) pathway. **b**, The glutamine synthetase-glutamate synthase (GS-GOGAT) pathway. ATP; Adenosine triphosphate. ADP; Adenosine diphosphate. Adapted from Charteris (2019).

**Supplementary Figure 2. Visualisation of percentage applied ^15^N incorporation (% ^15^N_A_ incorporation) and percentage retained ^15^N incorporation (%^15^N_R_ incorporation).** Excess moles ^15^N = moles ^15^N above natural abundance (= moles N × atom fraction excess). THAAs; Total hydrolysable amino acids.

**Supplementary Figure 3. Fig. 1 split into individual figures for clarity - time-course plots of AA *Δ*^15^N values revealing ^15^N assimilation into individual AAs in the six treatments. a**, RM-^15^NO_3_^-^. **b**, WA-^15^NO_3_^-^ (error bars at *t*=16 and 32 days are coloured to aid differentiation). **c**, RM-^15^NH_4_^+^. **d**, WA-^15^NH_4_^+^. **e**, RM-^15^N-U. **f**, WA-^15^N-U. Error bars are ± SE (n=3).

Supplementary Tables

**Supplementary Table 1. Mean *t*=0 and incubation bulk soil *δ*^15^N values and % ^15^N retentions for the six 32-day incubation experiments conducted.**

|  | *t=*0 | |  | Overall incubation means | | | | |
| --- | --- | --- | --- | --- | --- | --- | --- | --- |
|  | Mean *δ*^15^N value /‰ | SE | *n* | Mean *δ*^15^N value /‰ | SE | % ^15^N Retention /% | SE | *n* |
| RM-^15^NO_3_^-^ | 4.47 | 0.04 | 6 | 36.81 | 1.09 | 84 | 3 | 30 |
| RM-^15^NH_4_^+^ | 4.47 | 0.04 | 6 | 85.43 | 1.25 | 105 | 2 | 30 |
| RM-^15^N-U | 4.99 | 0.02 | 3 | 131.24 | 1.31 | 97 | 1 | 12 |
| WA-^15^NO_3_^-^ | 5.83 | 0.06 | 6 | 31.33 | 2.48 | 57 | 5 | 30 |
| WA-^15^NH_4_^+^ | 5.83 | 0.06 | 6 | 96.74 | 1.54 | 110 | 2 | 30 |
| WA-^15^N-U | 6.38 | 0.11 | 3 | 115.47 | 3.24 | 81 | 2 | 12 |

**Supplementary Table 2. Bulk soil % TC and % TN contents for each incubation experiment.** Bulk soil % TC contents are summarised as average values for all replicates at every time point (including *t*=0 pre-incubation values) for each treatment alongside associated SEs. Bulk soil % TN contents are displayed for every time point sampled in each incubation experiment (n=3; bars, ‘―’ indicate no samples were incubated for this time period) alongside mean values (for all replicates at every time point, including *t*=0 pre-incubation values) and their associated SEs.

|  | % TC | | | % TN | | | | | | | | | | | | | |
| --- | --- | --- | --- | --- | --- | --- | --- | --- | --- | --- | --- | --- | --- | --- | --- | --- | --- |
|  |  |  |  | Time /days | | | | | | | | | | | |  |  |
|  | Mean % TC | SE | n = | 0 | 0.0625 | 0.125 | 0.25 | 0.5 | 1 | 2 | 4 | 8 | 16 | 32 | 64 | Mean % TN | SE |
| RM-^15^NO_3_^-^ | 7.23 | 0.0578 | 36 | 0.63 | 0.67 | 0.65 | 0.67 | 0.66 | 0.65 | 0.67 | 0.66 | 0.66 | 0.68 | 0.69 | ― | 0.66 | 0.0039 |
| RM-^15^NH_4_^+^ | 7.21 | 0.0431 | 36 | 0.63 | 0.60 | 0.62 | 0.62 | 0.62 | 0.56 | 0.55 | 0.67 | 0.69 | 0.64 | 0.69 | ― | 0.63 | 0.0081 |
| RM-^15^N-U | 6.94 | 0.0488 | 15 | 0.64 | ― | 0.65 | ― | ― | ― | 0.64 | ― | ― | 0.67 | 0.67 | ― | 0.65 | 0.0050 |
| WA-^15^NO_3_^-^ | 7.33 | 0.0346 | 36 | 0.45 | 0.48 | 0.48 | 0.47 | 0.48 | 0.47 | 0.48 | 0.47 | 0.47 | 0.48 | 0.46 | ― | 0.47 | 0.0026 |
| WA-^15^NH_4_^+^ | 7.57 | 0.0216 | 36 | 0.45 | 0.49 | 0.49 | 0.48 | 0.47 | 0.48 | 0.49 | 0.49 | 0.47 | 0.48 | 0.49 | ― | 0.48 | 0.0040 |
| WA-^15^N-U | 5.66 | 0.0689 | 15 | 0.56 | ― | 0.58 | ― | ― | ― | 0.57 | ― | ― | 0.57 | 0.56 | ― | 0.57 | 0.0051 |

**Supplementary Table 3. Amino acid concentrations and the percentage contribution of total hydrolysable AA (THAA) N to the TN pool for the RM-^15^NO_3_^-^ incubation experiment at each time point analysed (n=3).**

|  |  | Time /days | | | | | | | | | | |  |  |  |
| --- | --- | --- | --- | --- | --- | --- | --- | --- | --- | --- | --- | --- | --- | --- | --- |
|  |  | 0 | 0.0625 | 0.125 | 0.25 | 0.5 | 1 | 2 | 4 | 8 | 16 | 32 |  | Mean | SE |
| Mean concentration /mg g^-1^ | Ala | 2.38 | 1.86 | 1.93 | 1.97 | 1.93 | 2.11 | 1.97 | 2.18 | 1.81 | 1.85 | 2.32 |  | 2.06 | 0.0558 |
|  | Asx | 1.61 | 1.92 | 1.99 | 2.02 | 2.01 | 1.83 | 2.19 | 1.82 | 1.55 | 1.56 | 1.15 |  | 1.77 | 0.0660 |
|  | Glx | 1.59 | 1.78 | 1.85 | 1.73 | 1.81 | 1.83 | 1.92 | 1.80 | 1.51 | 1.57 | 1.13 |  | 1.68 | 0.0551 |
|  | Gly | 1.83 | 1.27 | 1.39 | 1.38 | 1.36 | 1.53 | 1.42 | 1.42 | 1.20 | 1.47 | 1.82 |  | 1.49 | 0.0503 |
|  | Hyp | 0.12 | 0.12 | 0.12 | 0.12 | 0.12 | 0.14 | 0.12 | 0.13 | 0.11 | 0.10 | 0.12 |  | 0.12 | 0.0028 |
|  | Ile | 0.38 | 0.49 | 0.49 | 0.39 | 0.35 | 0.25 | 0.34 | 0.39 | 0.34 | 0.26 | 0.37 |  | 0.37 | 0.015 |
|  | Leu | 1.02 | 1.07 | 1.11 | 1.02 | 0.97 | 0.95 | 0.95 | 1.07 | 0.85 | 0.91 | 0.87 |  | 0.98 | 0.017 |
|  | Lys | 0.48 | 0.34 | 0.41 | 0.57 | 0.46 | 0.39 | 0.40 | 0.46 | 0.53 | 0.39 | 0.60 |  | 0.44 | 0.025 |
|  | Met | 0.07 | 0.13 | 0.12 | 0.12 | 0.10 | 0.09 | 0.10 | 0.07 | 0.06 | 0.08 | 0.06 |  | 0.09 | 0.004 |
|  | Phe | 0.48 | 0.58 | 0.59 | 0.59 | 0.54 | 0.56 | 0.43 | 0.51 | 0.37 | 0.49 | 0.41 |  | 0.50 | 0.015 |
|  | Pro | 1.23 | 1.11 | 1.14 | 1.08 | 1.10 | 1.23 | 1.12 | 1.22 | 1.01 | 0.97 | 1.23 |  | 1.14 | 0.0261 |
|  | Ser | 0.89 | 0.89 | 0.95 | 1.12 | 1.05 | 0.90 | 1.00 | 0.98 | 0.87 | 0.81 | 0.85 |  | 0.93 | 0.026 |
|  | Thr | 0.73 | 0.82 | 0.90 | 0.97 | 0.88 | 0.68 | 0.87 | 0.86 | 0.77 | 0.67 | 0.65 |  | 0.80 | 0.027 |
|  | Tyr | 0.22 | 0.34 | 0.31 | 0.37 | 0.34 | 0.34 | 0.23 | 0.26 | 0.19 | 0.25 | 0.21 |  | 0.27 | 0.012 |
|  | Val | 0.75 | 0.72 | 0.77 | 0.73 | 0.63 | 0.44 | 0.63 | 0.76 | 0.67 | 0.52 | 0.62 |  | 0.67 | 0.026 |
|  | THAA | 13.8 | 13.4 | 14.1 | 14.2 | 13.6 | 13.3 | 13.7 | 13.9 | 11.7 | 11.9 | 12.4 |  | 13.3 | 0.245 |
| % THAA N of TN | | 28.8 | 25.0 | 27.1 | 26.6 | 26.3 | 26.1 | 25.8 | 27.0 | 22.6 | 22.6 | 24.2 |  | 25.9 | 0.523 |

**Supplementary Table 4. Amino acid concentrations and the percentage contribution of THAA N to the TN pool for the RM-^15^NH_4_^+^ incubation experiment at each time point analysed (n=3).**

|  |  | Time /days | | | | | | | | | | |  |  |  |
| --- | --- | --- | --- | --- | --- | --- | --- | --- | --- | --- | --- | --- | --- | --- | --- |
|  |  | 0 | 0.0625 | 0.125 | 0.25 | 0.5 | 1 | 2 | 4 | 8 | 16 | 32 |  | Mean | SE |
| Mean concentration /mg g^-1^ | Ala | 2.38 | 1.92 | 1.79 | 1.84 | 1.91 | 1.87 | 1.93 | 1.90 | 2.02 | 2.11 | 2.09 |  | 2.03 | 0.0453 |
|  | Asx | 1.61 | 2.21 | 1.80 | 1.82 | 2.01 | 1.94 | 2.10 | 2.00 | 2.11 | 2.08 | 2.03 |  | 1.95 | 0.0512 |
|  | Glx | 1.59 | 2.02 | 1.81 | 1.82 | 1.88 | 1.81 | 1.89 | 1.83 | 1.92 | 1.89 | 1.88 |  | 1.83 | 0.0440 |
|  | Gly | 1.83 | 1.26 | 1.14 | 1.41 | 1.34 | 1.33 | 1.52 | 1.37 | 1.49 | 1.49 | 1.44 |  | 1.46 | 0.0435 |
|  | Hyp | 0.12 | 0.12 | 0.11 | 0.12 | 0.12 | 0.11 | 0.12 | 0.11 | 0.13 | 0.13 | 0.13 |  | 0.12 | 0.0024 |
|  | Ile | 0.38 | 0.61 | 0.54 | 0.43 | 0.46 | 0.52 | 0.55 | 0.45 | 0.48 | 0.48 | 0.47 |  | 0.48 | 0.014 |
|  | Leu | 1.02 | 1.16 | 1.15 | 1.05 | 1.07 | 1.05 | 1.09 | 1.03 | 1.09 | 1.13 | 1.12 |  | 1.09 | 0.0119 |
|  | Lys | 0.48 | 0.41 | 0.27 | 0.27 | 0.25 | 0.34 | 0.48 | 0.40 | 0.51 | 0.59 | 0.55 |  | 0.42 | 0.029 |
|  | Phe | 0.07 | 0.12 | 0.12 | 0.09 | 0.13 | 0.10 | 0.08 | 0.13 | 0.12 | 0.11 | 0.13 |  | 0.1 | 0.005 |
|  | Pro | 0.48 | 0.55 | 0.58 | 0.45 | 0.46 | 0.46 | 0.48 | 0.54 | 0.60 | 0.59 | 0.64 |  | 0.53 | 0.014 |
|  | Ser | 1.23 | 1.14 | 1.10 | 1.19 | 1.14 | 1.09 | 1.14 | 1.07 | 1.14 | 1.18 | 1.20 |  | 1.16 | 0.0197 |
|  | Thr | 0.89 | 1.02 | 0.85 | 0.84 | 0.94 | 0.92 | 1.04 | 0.95 | 1.07 | 1.12 | 1.05 |  | 0.97 | 0.023 |
|  | Tyr | 0.73 | 1.07 | 0.89 | 0.87 | 0.90 | 0.86 | 0.96 | 0.92 | 0.99 | 1.01 | 0.93 |  | 0.90 | 0.025 |
|  | Val | 0.22 | 0.29 | 0.29 | 0.23 | 0.24 | 0.24 | 0.26 | 0.30 | 0.36 | 0.35 | 0.35 |  | 0.28 | 0.0099 |
|  | Val | 0.75 | 0.95 | 0.80 | 0.76 | 0.72 | 0.83 | 0.90 | 0.78 | 0.82 | 0.83 | 0.78 |  | 0.81 | 0.022 |
|  | THAA | 13.8 | 14.9 | 13.2 | 13.2 | 13.6 | 13.5 | 14.5 | 13.8 | 14.8 | 15.1 | 14.8 |  | 14.1 | 0.197 |
| % THAA N of TN | | 28.8 | 30.5 | 26.4 | 26.5 | 27.5 | 30.3 | 33.5 | 25.9 | 27.2 | 29.9 | 26.8 |  | 28.5 | 0.555 |

**Supplementary Table 5. Amino acid concentrations and the percentage contribution of THAA N to the TN pool for RM-^15^N-U incubation experiment at each time point analysed (n=3).**

|  |  | Time /days | | | | |  |  |  |
| --- | --- | --- | --- | --- | --- | --- | --- | --- | --- |
|  |  | 0 | 0.125 | 2 | 16 | 32 |  | Mean | SE |
| Mean concentration /mg g^-1^ | Ala | 1.14 | 1.42 | 1.34 | 1.32 | 1.34 |  | 1.31 | 0.0340 |
|  | Asx | 1.60 | 1.55 | 1.42 | 1.42 | 1.63 |  | 1.53 | 0.0479 |
|  | Glx | 1.84 | 1.83 | 1.72 | 1.66 | 1.84 |  | 1.78 | 0.0381 |
|  | Gly | 0.92 | 1.43 | 1.32 | 1.29 | 1.31 |  | 1.25 | 0.056 |
|  | Hyp | 0.10 | 0.14 | 0.13 | 0.13 | 0.13 |  | 0.13 | 0.0048 |
|  | Ile | 0.47 | 0.22 | 0.25 | 0.22 | 0.25 |  | 0.28 | 0.027 |
|  | Leu | 0.97 | 0.85 | 0.83 | 0.83 | 0.85 |  | 0.87 | 0.017 |
|  | Lys | 0.21 | 0.37 | 0.35 | 0.32 | 0.37 |  | 0.32 | 0.019 |
|  | Phe | 0.54 | 0.44 | 0.45 | 0.45 | 0.48 |  | 0.47 | 0.011 |
|  | Pro | 0.95 | 1.20 | 1.16 | 1.11 | 1.10 |  | 1.10 | 0.034 |
|  | Ser | 0.71 | 0.77 | 0.71 | 0.71 | 0.79 |  | 0.74 | 0.022 |
|  | Thr | 1.06 | 0.81 | 0.78 | 0.78 | 0.88 |  | 0.86 | 0.039 |
|  | Tyr | 0.24 | 0.24 | 0.25 | 0.23 | 0.25 |  | 0.24 | 0.0037 |
|  | Val | 0.94 | 0.49 | 0.53 | 0.48 | 0.54 |  | 0.60 | 0.049 |
|  | THAA | 11.7 | 11.8 | 11.2 | 10.9 | 11.8 |  | 11.5 | 0.214 |
| % THAA N of TN | | 22.2 | 22.9 | 22.0 | 20.6 | 21.8 |  | 21.9 | 0.387 |

**Supplementary Table 6. Amino acid concentrations and the percentage contribution of THAA N to the TN pool for WA-^15^NO_3_^-^ incubation experiment at each time point analysed (n=3).**

|  |  | Time /days | | | | | | |  |  |  |
| --- | --- | --- | --- | --- | --- | --- | --- | --- | --- | --- | --- |
|  |  | 0 | 0.125 | 0.5 | 2 | 4 | 16 | 32 |  | Mean | SE |
| Mean concentration /mg g^-1^ | Ala | 0.86 | 1.00 | 0.91 | 0.84 | 0.96 | 0.86 | 0.86 |  | 0.89 | 0.019 |
|  | Asx | 1.25 | 2.28 | 2.32 | 1.84 | 2.34 | 2.30 | 2.19 |  | 1.97 | 0.108 |
|  | Glx | 1.23 | 1.72 | 1.72 | 1.49 | 1.76 | 1.58 | 1.53 |  | 1.53 | 0.0500 |
|  | Gly | 1.01 | 1.18 | 1.17 | 1.14 | 1.29 | 0.96 | 1.02 |  | 1.10 | 0.030 |
|  | Hyp | 0.09 | 0.13 | 0.14 | 0.10 | 0.13 | 0.12 | 0.11 |  | 0.11 | 0.004 |
|  | Ile | 0.21 | 0.23 | 0.24 | 0.18 | 0.20 | 0.29 | 0.23 |  | 0.22 | 0.012 |
|  | Leu | 0.53 | 0.64 | 0.56 | 0.59 | 0.59 | 0.61 | 0.58 |  | 0.58 | 0.0094 |
|  | Lys | 0.23 | 0.37 | 0.36 | 0.30 | 0.34 | 0.47 | 0.40 |  | 0.34 | 0.025 |
|  | Phe | 0.25 | 0.27 | 0.25 | 0.24 | 0.24 | 0.35 | 0.33 |  | 0.27 | 0.010 |
|  | Pro | 0.73 | 0.84 | 0.76 | 0.72 | 0.78 | 0.71 | 0.68 |  | 0.74 | 0.016 |
|  | Ser | 0.36 | 0.68 | 0.62 | 0.49 | 0.64 | 0.67 | 0.64 |  | 0.56 | 0.031 |
|  | Thr | 0.40 | 0.73 | 0.60 | 0.50 | 0.70 | 0.89 | 0.76 |  | 0.62 | 0.041 |
|  | Tyr | 0.09 | 0.11 | 0.12 | 0.10 | 0.11 | 0.14 | 0.13 |  | 0.11 | 0.004 |
|  | Val | 0.38 | 0.50 | 0.44 | 0.33 | 0.39 | 0.67 | 0.54 |  | 0.45 | 0.028 |
|  | THAA | 7.6 | 10.7 | 10.2 | 8.9 | 10.5 | 10.6 | 10.0 |  | 9.5 | 0.91 |
| % THAA N of TN | | 21.6 | 27.5 | 26.3 | 23.1 | 28.0 | 27.3 | 26.9 |  | 25.3 | 0.719 |

**Supplementary Table 7. Amino acid concentrations and the percentage contribution of THAA N to the TN pool for WA-^15^NH_4_^+^ incubation experiment at each time point analysed (n=3).**

|  |  | Time /days | | | | | | |  |  |  |
| --- | --- | --- | --- | --- | --- | --- | --- | --- | --- | --- | --- |
|  |  | 0 | 0.0625 | 0.125 | 0.5 | 2 | 8 | 32 |  | Mean | SE |
| Mean concentration /mg g^-1^ | Ala | 0.86 | 0.78 | 0.85 | 0.93 | 0.84 | 0.87 | 0.76 |  | 0.84 | 0.015 |
|  | Asx | 1.25 | 2.13 | 1.96 | 2.31 | 2.06 | 2.42 | 2.04 |  | 1.93 | 0.0954 |
|  | Glx | 1.23 | 1.53 | 1.45 | 1.69 | 1.50 | 1.72 | 1.54 |  | 1.49 | 0.0421 |
|  | Gly | 1.01 | 0.96 | 1.04 | 1.15 | 1.03 | 1.08 | 0.93 |  | 1.03 | 0.019 |
|  | Hyp | 0.09 | 0.11 | 0.11 | 0.13 | 0.11 | 0.12 | 0.11 |  | 0.11 | 0.003 |
|  | Ile | 0.21 | 0.20 | 0.17 | 0.26 | 0.22 | 0.24 | 0.22 |  | 0.21 | 0.0089 |
|  | Leu | 0.53 | 0.57 | 0.55 | 0.62 | 0.58 | 0.62 | 0.59 |  | 0.57 | 0.0084 |
|  | Lys | 0.23 | 0.31 | 0.26 | 0.38 | 0.31 | 0.35 | 0.31 |  | 0.30 | 0.013 |
|  | Phe | 0.25 | 0.26 | 0.24 | 0.33 | 0.25 | 0.35 | 0.37 |  | 0.29 | 0.010 |
|  | Pro | 0.73 | 0.68 | 0.68 | 0.77 | 0.69 | 0.72 | 0.67 |  | 0.71 | 0.012 |
|  | Ser | 0.36 | 0.61 | 0.58 | 0.70 | 0.64 | 0.71 | 0.60 |  | 0.57 | 0.029 |
|  | Thr | 0.40 | 0.69 | 0.63 | 0.88 | 0.77 | 0.85 | 0.73 |  | 0.67 | 0.039 |
|  | Tyr | 0.09 | 0.09 | 0.09 | 0.14 | 0.12 | 0.14 | 0.15 |  | 0.11 | 0.006 |
|  | Val | 0.38 | 0.46 | 0.42 | 0.62 | 0.51 | 0.55 | 0.49 |  | 0.48 | 0.020 |
|  | THAA | 7.6 | 9.4 | 9.1 | 10.9 | 9.6 | 10.7 | 9.5 |  | 9.3 | 0.27 |
| % THAA N of TN | | 21.6 | 23.7 | 22.8 | 29.1 | 24.4 | 28.1 | 23.7 |  | 24.4 | 0.720 |

**Supplementary Table 8. Amino acid concentrations and the percentage contribution of THAA N to the TN pool for WA-^15^N-U incubation experiment at each time point analysed (n=3).**

|  |  | Time /days | | | | |  |  |  |
| --- | --- | --- | --- | --- | --- | --- | --- | --- | --- |
|  |  | 0 | 0.125 | 2 | 16 | 32 |  | Mean | SE |
| Mean concentration /mg g^-1^ | Ala | 1.08 | 1.17 | 1.15 | 1.20 | 1.14 |  | 1.15 | 0.0241 |
|  | Asx | 1.57 | 1.36 | 1.72 | 1.25 | 1.60 |  | 1.50 | 0.0730 |
|  | Glx | 1.54 | 1.51 | 1.77 | 1.45 | 1.69 |  | 1.59 | 0.0591 |
|  | Gly | 1.03 | 1.15 | 1.16 | 1.19 | 1.11 |  | 1.13 | 0.0290 |
|  | Hyp | 0.15 | 0.17 | 0.18 | 0.19 | 0.19 |  | 0.18 | 0.0061 |
|  | Ile | 0.25 | 0.20 | 0.24 | 0.17 | 0.23 |  | 0.22 | 0.011 |
|  | Leu | 0.76 | 0.72 | 0.76 | 0.70 | 0.75 |  | 0.74 | 0.013 |
|  | Lys | 0.40 | 0.29 | 0.33 | 0.27 | 0.23 |  | 0.30 | 0.027 |
|  | Phe | 0.41 | 0.37 | 0.39 | 0.37 | 0.36 |  | 0.38 | 0.0086 |
|  | Pro | 0.82 | 0.94 | 0.91 | 1.03 | 0.99 |  | 0.94 | 0.030 |
|  | Ser | 0.74 | 0.58 | 0.73 | 0.60 | 0.72 |  | 0.68 | 0.028 |
|  | Thr | 0.78 | 0.63 | 0.79 | 0.57 | 0.77 |  | 0.71 | 0.036 |
|  | Tyr | 0.20 | 0.18 | 0.21 | 0.19 | 0.20 |  | 0.20 | 0.0044 |
|  | Val | 0.53 | 0.43 | 0.52 | 0.39 | 0.50 |  | 0.47 | 0.020 |
|  | THAA | 11.1 | 10.3 | 11.5 | 10.1 | 10.9 |  | 10.8 | 0.297 |
| % THAA N of TN | | 23.0 | 21.0 | 23.6 | 21.1 | 23.2 |  | 22.4 | 0.644 |

**Supplementary Table 9. Percentage variance accounted for (± SE) of simple exponential regressions^1^ fitted to time-course AA *Δ*^15^N data.** Percentages below 50% are in grey to highlight less reliable fits, a dash (-) indicates the residual variance exceeded the variance of the response variate.

|  | % variance accounted for /% | | | | | | | | | | | | |
| --- | --- | --- | --- | --- | --- | --- | --- | --- | --- | --- | --- | --- | --- |
|  |  | RM-^15^NO_3_^-^ | SE | WA-^15^NO_3_^-^ | SE | RM-^15^NH_4_^+^ | SE | WA-^15^NH_4_^+^ | SE | RM-^15^N-U | SE | WA-^15^N-U | SE |
| Ala |  | 74.9 | 0.7 | 63.7 | 3.8 | 94.4 | 3.0 | 97.2 | 1.7 | 98.5 | 1.8 | 98.3 | 1.6 |
| Asx |  | 73.0 | 0.6 | 58.0 | 2.6 | 93.2 | 4.0 | 97.0 | 1.3 | 98.4 | 2.0 | 98.1 | 1.6 |
| Glx |  | 69.5 | 0.8 | 48.2 | 5.5 | 84.2^a^ | 11.9 | 75.6^b^ | 6.4 | 95.3^c^ | 6.1 | 73.5^d^ | 9.4 |
| Gly |  | 43.7 | 0.9 | 60.4 | 2.4 | 94.5 | 2.3 | 95.4 | 1.4 | 8.3 | 7.6 | 98.2 | 1.0 |
| Hyp |  | 11.1 | 1.8 | - | 1.2 | 34.7 | 2.8 | - | 0.9 | 23.8 | 0.5 | 9.2 | 1.2 |
| Ile |  | 60.8 | 1.4 | 56.6 | 4.8 | 94.0 | 4.4 | 96.3 | 2.3 | 66.5 | 9.6 | 98.2 | 2.2 |
| Leu |  | 52.3 | 1.1 | 54.8 | 6.0 | 95.1 | 4.1 | 97.0 | 2.7 | 98.6 | 2.5 | 98.7 | 1.9 |
| Lys |  | 36.3 | 1.3 | 65.6 | 3.3 | 95.2 | 2.6 | 94.9 | 1.9 | 97.8 | 1.7 | 96.8 | 1.6 |
| Phe |  | 25.0 | 2.0 | 60.9 | 4.0 | 93.8 | 3.6 | 96.4 | 2.0 | 99.1 | 1.5 | 98.9 | 1.4 |
| Pro |  | 23.9 | 0.9 | 60.6 | 3.5 | 93.9 | 2.8 | 97.2 | 1.5 | 98.4 | 1.4 | 98.5 | 1.2 |
| Ser |  | 74.0 | 0.5 | 62.8 | 3.2 | 94.4 | 2.8 | 96.7 | 1.7 | 98.2 | 1.7 | 98.9 | 1.2 |
| Thr |  | 46.4 | 1.0 | 59.1 | 3.3 | 91.5 | 3.7 | 96.3 | 1.6 | 98.2 | 1.5 | 99.2 | 0.9 |
| Tyr |  | - | 2.2 | 55.2 | 4.7 | 89.5 | 4.8 | 96.4 | 2.1 | 96.8 | 2.7 | 94.3 | 2.4 |
| Val |  | 40.2 | 1.0 | 56.1 | 2.5 | 94.6 | 2.6 | 98.0 | 0.9 | 49.4 | 5.8 | 90.0 | 2.6 |

^1^ Critical exponential regressions were also performed for Glx in the ^15^NH_4_^+^ and ^15^N-U treatments with the following results: ^a^92.9 ± 8.0%; ^b^82.2 ± 5.4%; ^c^99.8 ± 1.3%; ^d^93.1 ± 4.8%.

**Supplementary Table 10. Amino acid plateau *Δ*^15^N values (± SE) determined by simple exponential regressions^1^.** A dash (-) indicates an SE could not be determined and should therefore be considered large.

|  |  | Plateau *Δ*^15^N values /‰ | | | | | | | | | | | |
| --- | --- | --- | --- | --- | --- | --- | --- | --- | --- | --- | --- | --- | --- |
|  |  | RM-^15^NO_3_^-^ | SE | WA-^15^NO_3_^-^ | SE | RM-^15^NH_4_^+^ | SE | WA-^15^NH_4_^+^ | SE | RM-^15^N-U | SE | WA-^15^N-U | SE |
| Ala |  | 3.0 | 0.35 | 14.1 | 3.7 | 29.7 | 1.0 | 23.5 | 0.70 | 33.1 | 1.2 | 30.2 | 0.67 |
| Asx |  | 2.9 | 0.69 | 9.5 | 5.2 | 35.7 | 1.3 | 16.2 | 0.53 | 33.7 | 1.1 | 27.4 | 0.64 |
| Glx |  | 2.8 | 0.55 | 14.6 | 4.0 | 75.1^a^ | 3.1 | 38.8^b^ | - | 64.5^c^ | 2.6 | 50.5^d^ | - |
| Gly |  | 2.2 | 0.41 | 7.5 | 1.6 | 23.4 | 0.76 | 14.5 | 0.64 | 21.3 | 4.2 | 17.6 | 0.40 |
| Hyp |  | 2.3 | 0.58 | -1.1 | - | 6.9 | - | -1.4 | - | 1.5 | - | -1.8 | 0.47 |
| Ile |  | 3.8 | 0.71 | 14.7 | 3.3 | 42.5 | 1.5 | 29.6 | 1.0 | 44.0 | 4.4 | 37.9 | 0.90 |
| Leu |  | 4.5 | 4.3 | 19.5 | 10.0 | 43.4 | 1.4 | 35.9 | 1.2 | 46.5 | 1.4 | 40.2 | 0.77 |
| Lys |  | 4.6 | 1.6 | 11.8 | 1.9 | 28.2 | 0.88 | 20.6 | 0.94 | 25.5 | 0.91 | 19.7 | 0.67 |
| Phe |  | 4.8 | 7.1 | 17.3 | 10.9 | 34.4 | 1.2 | 24.9 | 0.95 | 34.7 | 1.0 | 30.8 | 0.56 |
| Pro |  | 1.3 | 1.0 | 11.6 | 4.1 | 27.1 | 1.0 | 21.8 | 0.80 | 24.3 | 0.88 | 22.9 | 0.48 |
| Ser |  | 2.4 | 0.42 | 11.7 | 2.9 | 27.8 | 0.92 | 20.9 | 0.71 | 28.1 | 1.1 | 26.5 | 0.49 |
| Thr |  | 2.7 | 1.2 | 10.6 | 3.9 | 29.0 | 1.2 | 19.2 | 0.74 | 26.0 | 0.86 | 22.3 | 0.36 |
| Tyr |  | 0.1 | 1.3 | 14.8 | 3.6 | 31.9 | 1.6 | 27.5 | 1.0 | 35.2 | 1.4 | 26.2 | 1.0 |
| Val |  | 1.7 | 0.53 | 6.6 | 1.5 | 25.7 | 0.89 | 14.0 | 0.39 | 21.0 | 4.2 | 17.7 | 1.1 |

^1^ Critical exponential regressions were also performed for Glx in the ^15^NH_4_^+^ and ^15^N-U treatments with the following results: ^a^64.5 ± 2.9‰; ^b^35.1 ± 2.2‰; ^c^48.3 ± 1.8‰; ^d^*ca.* 44.2‰.

**Supplementary Table 11. Percentage variance accounted for (± SE) of simple exponential regressions^1^ of the % ^15^N_R_ incorporated into AAs over time.** Percentages below 50% are in grey to highlight less reliable fits, a dash (-) indicates the residual variance exceeded the variance of the response variate.

|  | % variance accounted for /% | | | | | | | | | | | | |
| --- | --- | --- | --- | --- | --- | --- | --- | --- | --- | --- | --- | --- | --- |
|  |  | RM-^15^NO_3_^-^ | SE | WA-^15^NO_3_^-^ | SE | RM-^15^NH_4_^+^ | SE | WA-^15^NH_4_^+^ | SE | RM-^15^N-U | SE | WA-^15^N-U | SE |
| Ala |  | 64.3 | 0.10 | 51.2 | 0.34 | 93.2 | 0.20 | 96.2 | 0.06 | 98.0 | 0.05 | 95.0 | 0.08 |
| Asx |  | 45.0 | 0.06 | 47.6 | 0.41 | 91.8 | 0.18 | 96.1 | 0.08 | 97.8 | 0.05 | 83.6 | 0.12 |
| Glx |  | 42.8 | 0.07 | 41.5 | 0.53 | 75.3^a^ | 0.56 | 80.1^b^ | 0.20 | 96.7^c^ | 0.10 | 52.5^d^ | 0.36 |
| Gly |  | 37.2 | 0.12 | 49.9 | 0.32 | 91.6 | 0.15 | 96.8 | 0.05 | - | 0.25 | 95.7 | 0.05 |
| Hyp |  | 3.0 | 0.01 | - | 0.02 | 34.1 | 0.01 | - | 0.00 | 1.2 | 0.00 | 6.5 | 0.00 |
| Ile |  | 47.3 | 0.03 | 43.8 | 0.09 | 88.0 | 0.06 | 91.7 | 0.02 | 68.8 | 0.03 | 85.6 | 0.02 |
| Leu |  | 28.9 | 0.06 | 49.1 | 0.22 | 93.4 | 0.11 | 97.2 | 0.04 | 98.5 | 0.03 | 97.0 | 0.04 |
| Lys |  | 34.1 | 0.05 | 37.7 | 0.24 | 77.7 | 0.12 | 88.4 | 0.04 | 91.2 | 0.03 | 78.5 | 0.04 |
| Phe |  | - | 0.05 | 50.3 | 0.07 | 95.7 | 0.03 | 97.2 | 0.01 | 98.6 | 0.01 | 96.8 | 0.01 |
| Pro |  | 11.0 | 0.06 | 52.7 | 0.19 | 91.4 | 0.09 | 97.7 | 0.03 | 98.1 | 0.02 | 97.0 | 0.03 |
| Ser |  | 62.3 | 0.03 | 51.6 | 0.19 | 91.0 | 0.10 | 95.9 | 0.04 | 97.7 | 0.02 | 87.5 | 0.06 |
| Thr |  | 27.9 | 0.04 | 48.3 | 0.21 | 83.0 | 0.12 | 94.2 | 0.05 | 97.0 | 0.02 | 83.2 | 0.05 |
| Tyr |  | - | 0.03 | 36.2 | 0.04 | 94.0 | 0.02 | 97.0 | 0.01 | 97.2 | 0.01 | 92.9 | 0.01 |
| Val |  | 29.7 | 0.04 | 43.7 | 0.13 | 85.5 | 0.08 | 90.8 | 0.03 | 55.6 | 0.04 | 75.6 | 0.04 |

^1^ Critical exponential regressions were also performed for Glx in the ^15^NH_4_^+^ and ^15^N-U treatments with the following results: ^a^86.2 ± 0.42%; ^b^83.5 ± 0.19%; ^c^98.1 ± 0.08%; ^d^93.1 ± 0.14%.

**Supplementary Table 12. Amino acid plateau % ^15^N_R_ incorporations (± SE) determined by simple exponential regressions^1^.** A dash (-) indicates an SE could not be determined and should therefore be considered large.

|  |  | Plateau % ^15^N_R_ incorporations /% | | | | | | | | | | | |
| --- | --- | --- | --- | --- | --- | --- | --- | --- | --- | --- | --- | --- | --- |
|  |  | RM-^15^NO_3_^-^ | SE | WA-^15^NO_3_^-^ | SE | RM-^15^NH_4_^+^ | SE | WA-^15^NH_4_^+^ | SE | RM-^15^N-U | SE | WA-^15^N-U | SE |
| Ala |  | 0.39 | 0.054 | 0.85 | 0.15 | 1.82 | 0.067 | 0.72 | 0.025 | 0.84 | 0.035 | 0.83 | 0.032 |
| Asx |  | 0.13 | 0.034 | 0.86 | 0.22 | 1.46 | 0.059 | 0.91 | 0.035 | 0.68 | 0.032 | 0.64 | 0.046 |
| Glx |  | 0.13 | 0.036 | 1.0 | 0.21 | 2.65^a^ | 0.15 | 1.34^b^ | - | 1.28^c^ | 0.043 | 1.25^d^ | - |
| Gly |  | 0.33 | 0.091 | 0.71 | 0.13 | 1.20 | 0.049 | 0.64 | 0.024 | 0.62 | 0.14 | 0.56 | 0.020 |
| Hyp |  | 0.01 | 0.003 | -0.01 | - | 0.02 | 0.002 | 0.00 | - | 0.00 | - | -0.01 | 0.001 |
| Ile |  | 0.05 | 0.012 | 0.19 | 0.038 | 0.40 | 0.019 | 0.17 | 0.009 | 0.13 | 0.013 | 0.13 | 0.009 |
| Leu |  | 0.15 | 0.19 | 0.51 | 0.13 | 0.97 | 0.035 | 0.56 | 0.019 | 0.50 | 0.016 | 0.47 | 0.014 |
| Lys |  | 0.24 | 0.17 | 0.51 | 0.11 | 0.56 | 0.046 | 0.31 | 0.023 | 0.21 | 0.017 | 0.15 | 0.015 |
| Phe |  | 0.07 | 0.19 | 0.21 | 0.077 | 0.34 | 0.011 | 0.19 | 0.007 | 0.17 | 0.007 | 0.14 | 0.004 |
| Pro |  | 0.09 | 0.14 | 0.44 | 0.10 | 0.72 | 0.032 | 0.42 | 0.014 | 0.39 | 0.015 | 0.42 | 0.013 |
| Ser |  | 0.10 | 0.018 | 0.46 | 0.079 | 0.75 | 0.032 | 0.44 | 0.017 | 0.35 | 0.017 | 0.36 | 0.023 |
| Thr |  | 0.06 | 0.032 | 0.46 | 0.12 | 0.63 | 0.037 | 0.43 | 0.022 | 0.32 | 0.016 | 0.27 | 0.021 |
| Tyr |  | 0.00 | 0.008 | 0.08 | 0.018 | 0.16 | 0.006 | 0.08 | 0.003 | 0.08 | 0.003 | 0.06 | - |
| Val |  | 0.05 | 0.019 | 0.25 | 0.062 | 0.47 | 0.027 | 0.21 | 0.014 | 0.17 | 0.040 | 0.15 | 0.015 |

^1^ Critical exponential regressions were also performed for Glx in the ^15^NH_4_^+^ and ^15^N-U treatments with the following results: ^a^2.2 ± 0.15‰; ^b^*ca.* 1.3‰; ^c^1.1 ± 0.08‰; ^d^1.0 ± 0.06‰.

**Supplementary Table 13. Percentage variance accounted for (± SE) of linear regressions fitted to time-course % ^15^N_R_ incorporation data (over the [sometimes very] approximately linear first 4, 0.5 and 2 days for the ^15^NO_3_^-^, ^15^NH_4_^+^ and ^15^N-U treatments, respectively).** Percentages below 50% are in grey to highlight less reliable fits, a dash (-) indicates the residual variance exceeded the variance of the response variate.

|  | % variance accounted for /% | | | | | | | | | | | | |
| --- | --- | --- | --- | --- | --- | --- | --- | --- | --- | --- | --- | --- | --- |
|  |  | RM-^15^NO_3_^-^ | SE | WA-^15^NO_3_^-^ | SE | RM-^15^NH_4_^+^ | SE | WA-^15^NH_4_^+^ | SE | RM-^15^N-U | SE | WA-^15^N-U | SE |
| Ala |  | 32.1 | 0.09 | 74.1 | 0.16 | 69.4 | 0.05 | 89.4 | 0.03 | 99.1 | 0.01 | 97.0 | 0.05 |
| Asx |  | 13.9 | 0.07 | 52.9 | 0.26 | 72.6 | 0.05 | 87.8 | 0.04 | 95.6 | 0.01 | 97.4 | 0.05 |
| Glx |  | 9.4 | 0.08 | 77.6 | 0.25 | 43.1 | 0.34 | 28.3 | 0.26 | 98.0 | 0.04 | 81.9 | 0.30 |
| Gly |  | 36.7 | 0.09 | 75.2 | 0.19 | 10.5 | 0.06 | 73.4 | 0.03 | - | 0.34 | 95.2 | 0.04 |
| Hyp |  | - | 0.01 | - | 0.02 | - | 0.01 | - | 0.00 | - | 0.00 | - | 0.00 |
| Ile |  | - | 0.02 | 52.4 | 0.05 | 47.3 | 0.01 | 87.8 | 0.01 | - | 0.04 | 97.1 | 0.01 |
| Leu |  | - | 0.07 | 58.2 | 0.11 | 77.2 | 0.02 | 97.8 | 0.01 | 96.7 | 0.01 | 99.0 | 0.02 |
| Lys |  | - | 0.04 | 38.6 | 0.13 | 22.9 | 0.01 | 33.8 | 0.01 | 64.8 | 0.01 | 95.5 | 0.02 |
| Phe |  | - | 0.06 | 71.6 | 0.01 | 62.4 | 0.01 | 94.0 | 0.00 | 94.8 | 0.00 | 98.6 | 0.01 |
| Pro |  | - | 0.07 | 56.6 | 0.11 | 38.5 | 0.02 | 78.8 | 0.02 | 94.1 | 0.01 | 98.0 | 0.02 |
| Ser |  | 20.2 | 0.03 | 76.6 | 0.09 | 71.3 | 0.02 | 95.2 | 0.01 | 93.0 | 0.01 | 98.3 | 0.02 |
| Thr |  | - | 0.05 | 46.8 | 0.13 | 65.2 | 0.03 | 86.3 | 0.01 | 79.8 | 0.01 | 98.9 | 0.01 |
| Tyr |  | - | 0.03 | 16.8 | 0.02 | 29.0 | 0.01 | 62.2 | 0.00 | 20.2 | 0.01 | 88.5 | 0.01 |
| Val |  | 0.9 | 0.04 | 53.0 | 0.06 | 36.7 | 0.02 | 86.0 | 0.01 | - | 0.05 | 92.6 | 0.02 |

**Supplementary Table 14. Zero order rate constants (± SE) from linear regressions performed over the (sometimes very) approximately linear first 4, 0.5 and 2 days for the ^15^NO_3_^-^, ^15^NH_4_^+^ and ^15^N-U treatments, respectively.** A dash (-) indicates an SE could not be determined and should therefore be considered large.

|  |  | Zero order rate constants of AA % ^15^N_R_ incorporations /% day^-1^ | | | | | | | | | | | |
| --- | --- | --- | --- | --- | --- | --- | --- | --- | --- | --- | --- | --- | --- |
|  |  | RM-^15^NO_3_^-^ | SE | WA-^15^NO_3_^-^ | SE | RM-^15^NH_4_^+^ | SE | WA-^15^NH_4_^+^ | SE | RM-^15^N-U | SE | WA-^15^N-U | SE |
| Ala |  | 0.048 | 0.010 | 0.16 | 0.021 | 0.48 | 0.051 | 0.42 | 0.033 | 0.072 | 0.002 | 0.34 | 0.016 |
| Asx |  | 0.016 | 0.008 | 0.13 | 0.033 | 0.41 | 0.047 | 0.38 | 0.039 | 0.062 | 0.004 | 0.34 | 0.016 |
| Glx |  | 0.018 | 0.010 | 0.28 | 0.033 | 2.6 | 0.34 | 2.0 | 0.29 | 0.30 | 0.012 | 0.85 | 0.087 |
| Gly |  | 0.056 | 0.012 | 0.18 | 0.024 | 0.33 | 0.062 | 0.21 | 0.035 | 0.072 | 0.098 | 0.22 | 0.013 |
| Hyp |  | 0.004 | 0.002 | -0.008 | 0.002 | 0.016 | 0.005 | -0.010 | 0.003 | -0.001 | 0.000 | -0.002 | 0.001 |
| Ile |  | 0.003 | 0.003 | 0.031 | 0.006 | 0.10 | 0.014 | 0.10 | 0.007 | 0.021 | 0.011 | 0.062 | 0.003 |
| Leu |  | -0.004 | 0.008 | 0.061 | 0.014 | 0.24 | 0.024 | 0.19 | 0.007 | 0.051 | 0.003 | 0.21 | 0.006 |
| Lys |  | 0.014 | 0.005 | 0.079 | 0.017 | 0.058 | 0.010 | 0.10 | 0.015 | 0.023 | 0.003 | 0.073 | 0.005 |
| Phe |  | 0.000 | 0.007 | 0.013 | 0.002 | 0.065 | 0.007 | 0.056 | 0.004 | 0.013 | 0.001 | 0.061 | 0.002 |
| Pro |  | 0.000 | 0.009 | 0.058 | 0.014 | 0.12 | 0.025 | 0.13 | 0.019 | 0.035 | 0.002 | 0.14 | 0.005 |
| Ser |  | 0.010 | 0.004 | 0.093 | 0.012 | 0.19 | 0.022 | 0.17 | 0.010 | 0.029 | 0.002 | 0.17 | 0.006 |
| Thr |  | 0.004 | 0.006 | 0.056 | 0.017 | 0.20 | 0.030 | 0.15 | 0.016 | 0.033 | 0.004 | 0.13 | 0.004 |
| Tyr |  | 0.000 | 0.004 | 0.010 | 0.003 | 0.012 | 0.006 | 0.024 | 0.003 | 0.010 | 0.002 | 0.028 | 0.002 |
| Val |  | 0.006 | 0.005 | 0.033 | 0.008 | 0.069 | 0.020 | 0.087 | 0.010 | 0.013 | 0.015 | 0.071 | 0.006 |

**Supplementary Table 15. First order rate constants (± SE) from simple exponential regressions^1^ of time-course % ^15^N_R_ incorporations into hydrolysable AAs.** A dash (-) indicates an SE could not be determined and should therefore be considered large.

|  |  | First order rate constants of AA % ^15^N_R_ incorporations /day^-1^ | | | | | | | | | | | |
| --- | --- | --- | --- | --- | --- | --- | --- | --- | --- | --- | --- | --- | --- |
|  |  | RM-^15^NO_3_^-^ | SE | WA-^15^NO_3_^-^ | SE | RM-^15^NH_4_^+^ | SE | WA-^15^NH_4_^+^ | SE | RM-^15^N-U | SE | WA-^15^N-U | SE |
| Ala |  | 0.86 | 0.050 | 0.76 | 0.11 | 0.58 | 0.041 | 0.58 | 0.040 | 0.88 | 0.020 | 0.44 | 0.069 |
| Asx |  | 0.87 | 0.086 | 0.85 | 0.11 | 0.52 | 0.049 | 0.62 | 0.042 | 0.89 | 0.019 | 0.20 | 0.19 |
| Glx |  | 0.85 | 0.11 | 0.60 | 0.22 | 0.14^a^ | 0.064 | 0.14^b^ | - | 0.72^c^ | 0.034 | 0.18^d^ | - |
| Gly |  | 0.91 | 0.057 | 0.68 | 0.16 | 0.55 | 0.047 | 0.68 | 0.035 | 0.85 | 0.14 | 0.48 | 0.057 |
| Hyp |  | 0.16 | 0.37 | 0.15 | - | 0.13 | 0.13 | 0.14 | - | 0.18 | - | 0.58 | 0.34 |
| Ile |  | 0.83 | 0.11 | 0.79 | 0.12 | 0.46 | 0.064 | 0.61 | 0.057 | 0.82 | 0.077 | 0.26 | 0.18 |
| Leu |  | 0.96 | 0.073 | 0.87 | 0.095 | 0.54 | 0.043 | 0.68 | 0.031 | 0.86 | 0.017 | 0.36 | 0.064 |
| Lys |  | 0.96 | 0.039 | 0.79 | 0.13 | 0.71 | 0.065 | 0.73 | 0.058 | 0.87 | 0.041 | 0.32 | 0.23 |
| Phe |  | 0.97 | 0.14 | 0.92 | 0.066 | 0.68 | 0.027 | 0.81 | 0.021 | 0.89 | 0.014 | 0.39 | 0.063 |
| Pro |  | 0.96 | 0.12 | 0.85 | 0.10 | 0.65 | 0.043 | 0.75 | 0.025 | 0.87 | 0.019 | 0.59 | 0.039 |
| Ser |  | 0.88 | 0.053 | 0.75 | 0.12 | 0.58 | 0.049 | 0.63 | 0.041 | 0.89 | 0.019 | 0.30 | 0.16 |
| Thr |  | 0.90 | 0.11 | 0.86 | 0.10 | 0.44 | 0.079 | 0.69 | 0.046 | 0.88 | 0.022 | 0.32 | 0.18 |
| Tyr |  | 1.1 | 0.85 | 0.84 | 0.11 | 0.66 | 0.036 | 0.78 | 0.024 | 0.86 | 0.023 | 0.18 | - |
| Val |  | 0.82 | 0.17 | 0.84 | 0.12 | 0.53 | 0.070 | 0.68 | 0.060 | 0.91 | 0.059 | 0.32 | 0.24 |

^1^ Critical exponential regressions were also performed for Glx in the ^15^NH_4_^+^ and ^15^N-U treatments with the following results: ^a^3.0 ± 0.33 day^-1^; ^b^*ca.* 1.1 day^-1^; ^c^0.22 ± 0.028 day^-1^; ^d^3.6 ± 0.66 day^-1^.

**Supplementary Table 16. Percentage variance explained (± SE) and plateau % ^15^N_R_ incorporations (± SE) determined by simple exponential regressions of the % ^15^N incorporated into the total hydrolysable AA (THAA) pool over time.** A dash (-) indicates an SE could not be determined and should therefore be considered large.

|  |  | % ^15^N_A_ in THAA | | | | | | | | | | | |
| --- | --- | --- | --- | --- | --- | --- | --- | --- | --- | --- | --- | --- | --- |
|  |  | RM-^15^NO_3_^-^ | SE | WA-^15^NO_3_^-^ | SE | RM-^15^NH_4_^+^ | SE | WA-^15^NH_4_^+^ | SE | RM-^15^N-U | SE | WA-^15^N-U | SE |
| % var. ex. (± SE) |  | 77.7 | 0.35 | 56.2 | 2.2 | 94.3 | 1.3 | 98.3 | 0.35 | 95.7 | 0.47 | 90.7 | 0.58 |
| Plateau % ^15^N (± SE) |  | 1.7 | 0.27 | 6.6 | 1.8 | 12.9 | 0.41 | 6.5 | 0.14 | 5.4 | 0.24 | 4.4 | 0.23 |
|  |  | % ^15^N_R_ in THAA | | | | | | | | | | | |
|  |  | RM-^15^NO_3_^-^ | SE | WA-^15^NO_3_^-^ | SE | RM-^15^NH_4_^+^ | SE | WA-^15^NH_4_^+^ | SE | RM-^15^N-U | SE | WA-^15^N-U | SE |
| % var. ex. (± SE) |  | 69.2 | 0.44 | 50.4 | 2.8 | 92.6 | 1.4 | 95.3 | 0.56 | 96.1 | 0.47 | 93.1 | 0.59 |
| Plateau % ^15^N (± SE) |  | 1.6 | 0.29 | 6.4 | 1.2 | 11.7 | 0.41 | 6.3 | 0.24 | 5.6 | 0.25 | 5.2 | - |
