# Supplementary figures and images for: The differential assimilation of nitrogen fertilizer compounds by soil microorganisms
Source: FEMS Microbiol Lett. 2024 Jun 7;371:fnae041. doi: 10.1093/femsle/fnae041 (PMC11223579; doi:10.1093/femsle/fnae041)

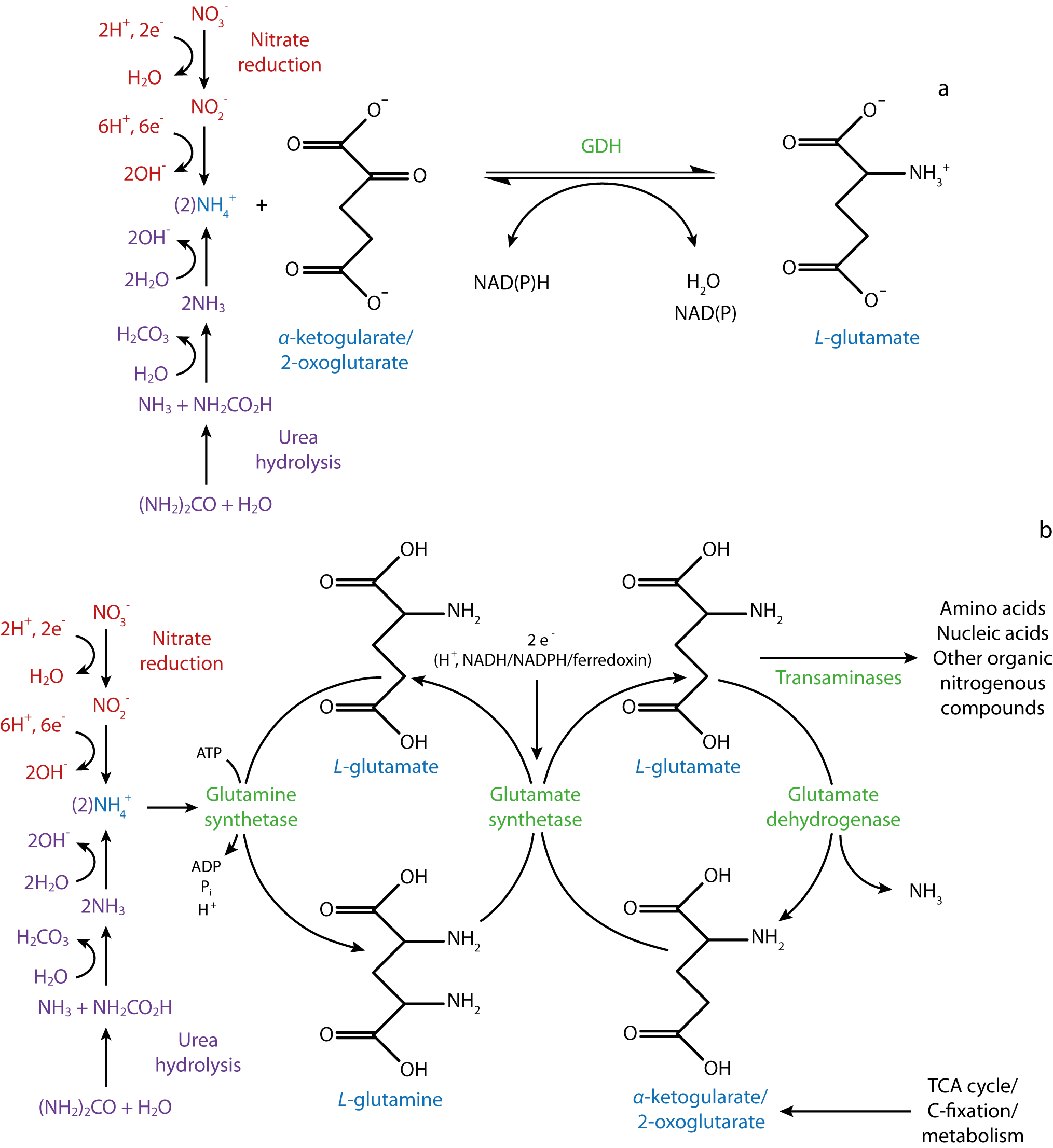

Supplement: fnae041_Supplemental_Files [file fnae041_supplemental_files.zip › S.Fig.1.tif]

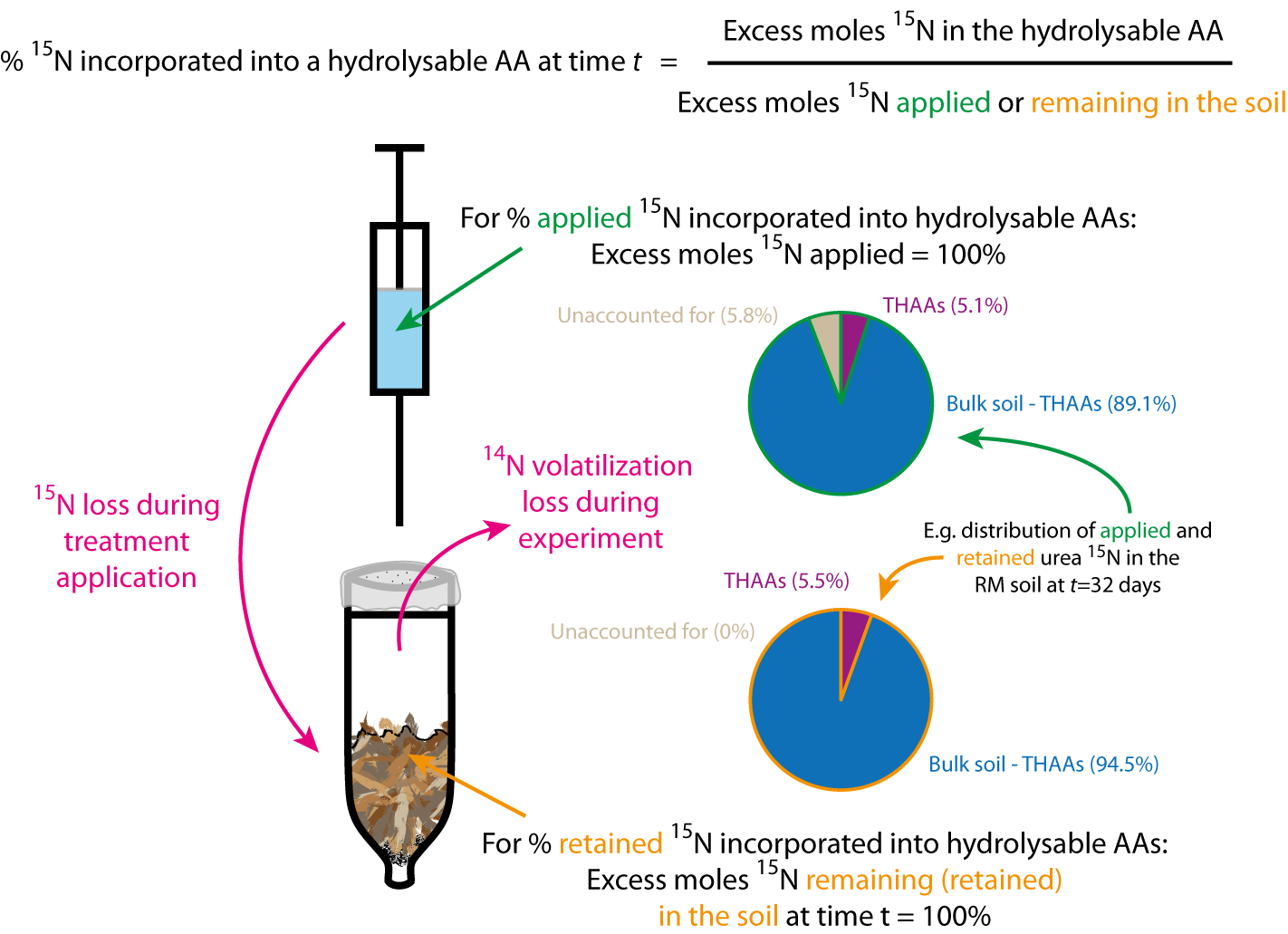

Supplement: fnae041_Supplemental_Files [file fnae041_supplemental_files.zip › S.Fig.2.tif]

**a**

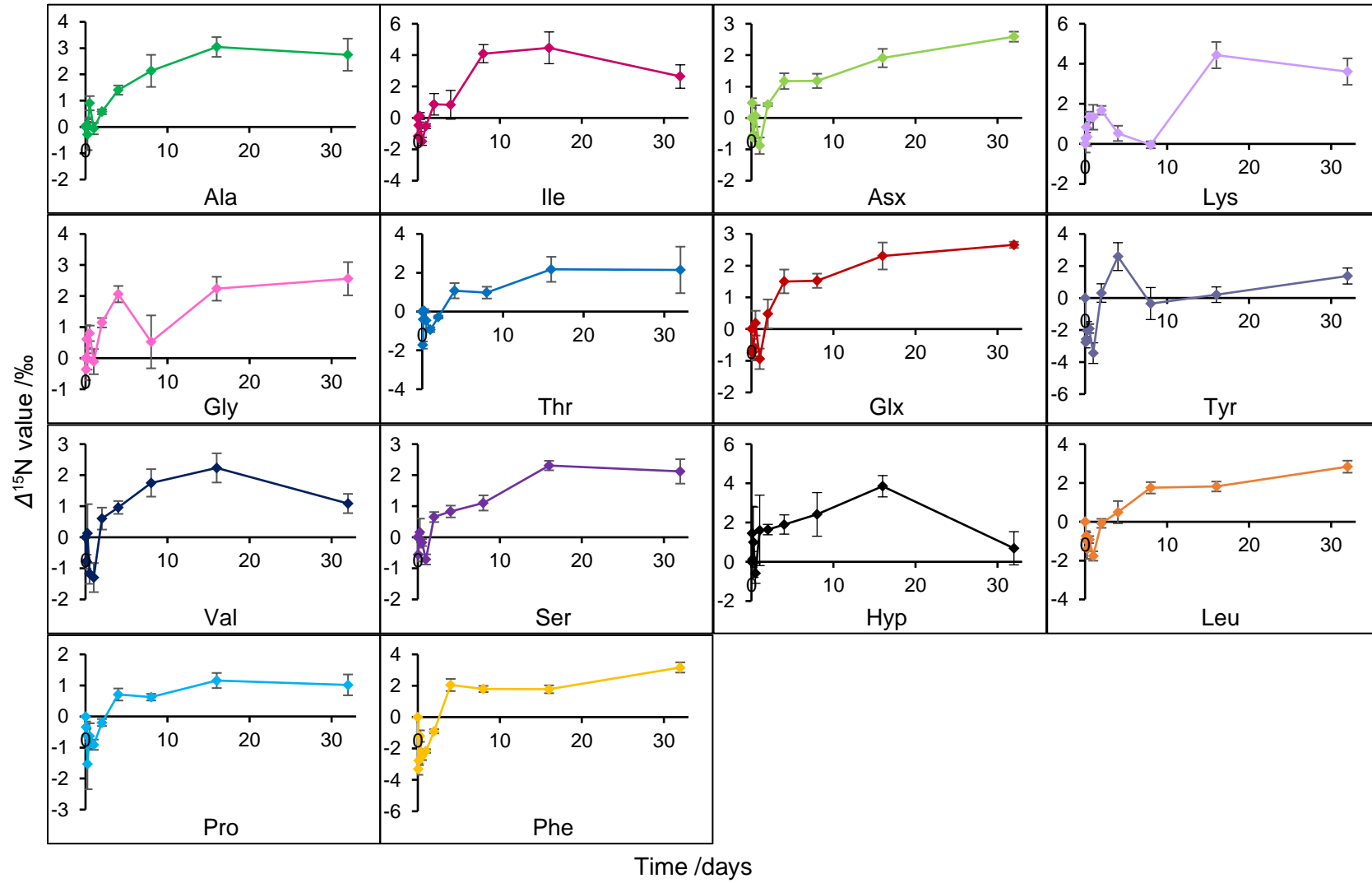

**b**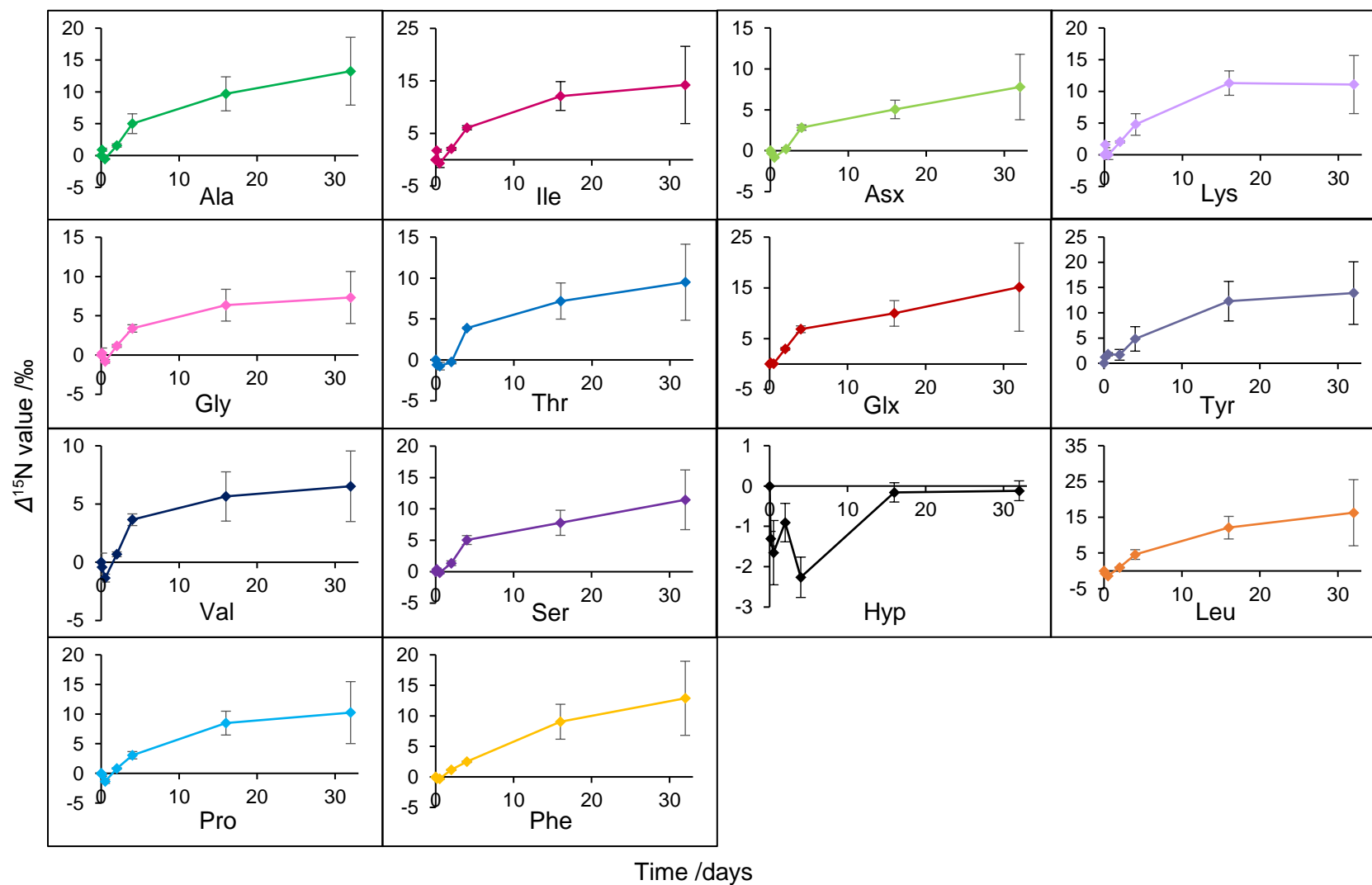

**c**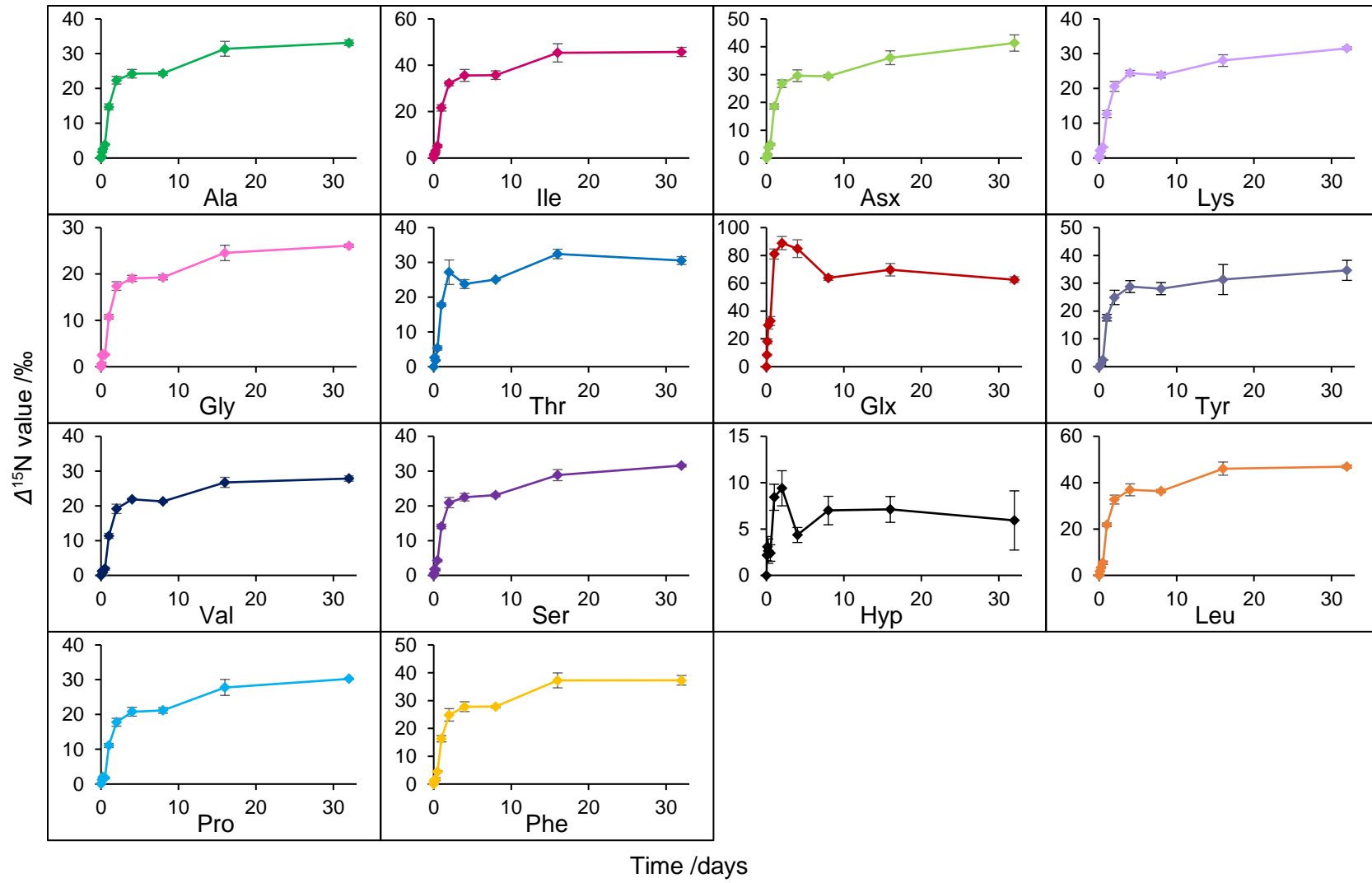

d

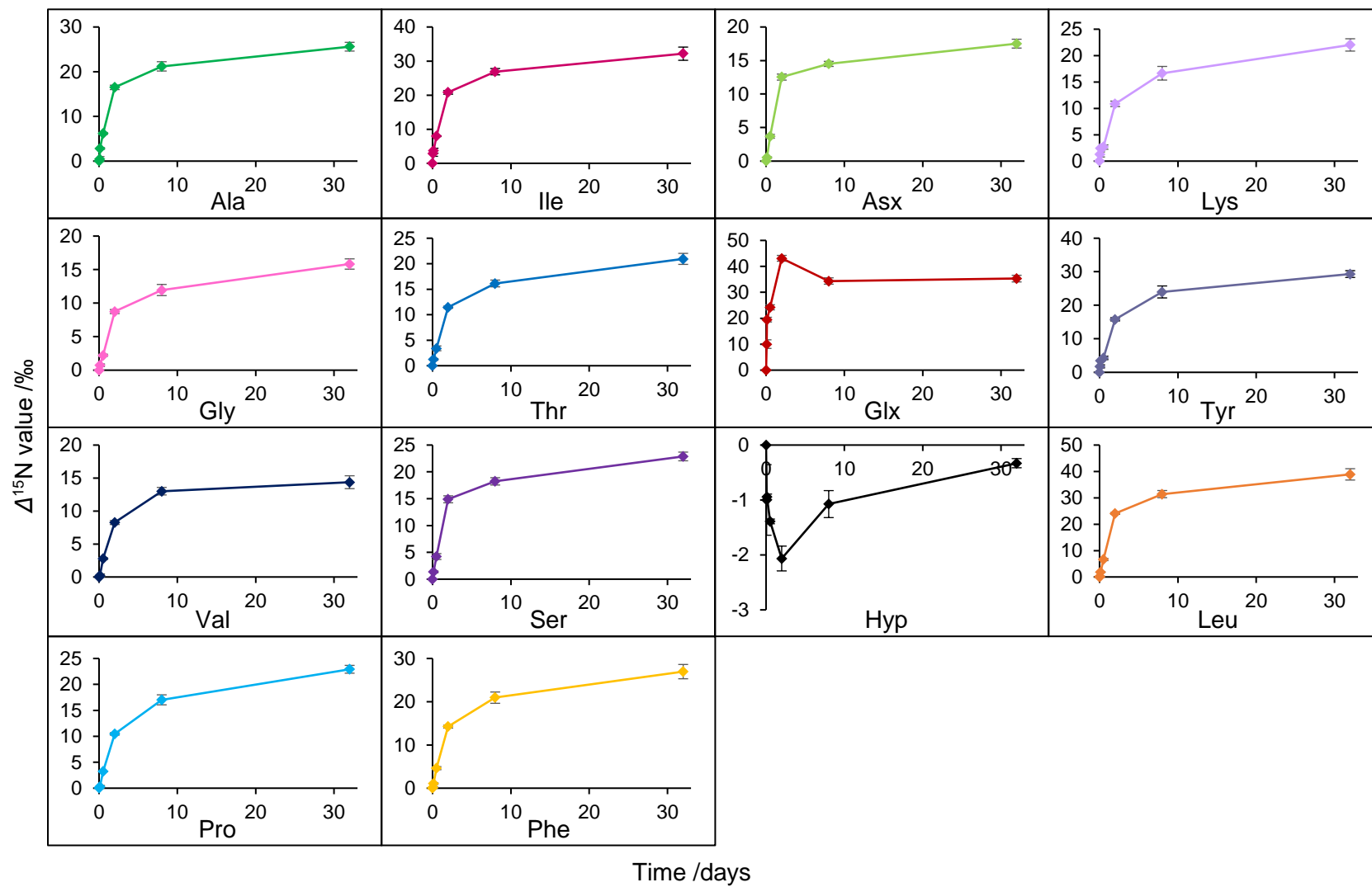

e

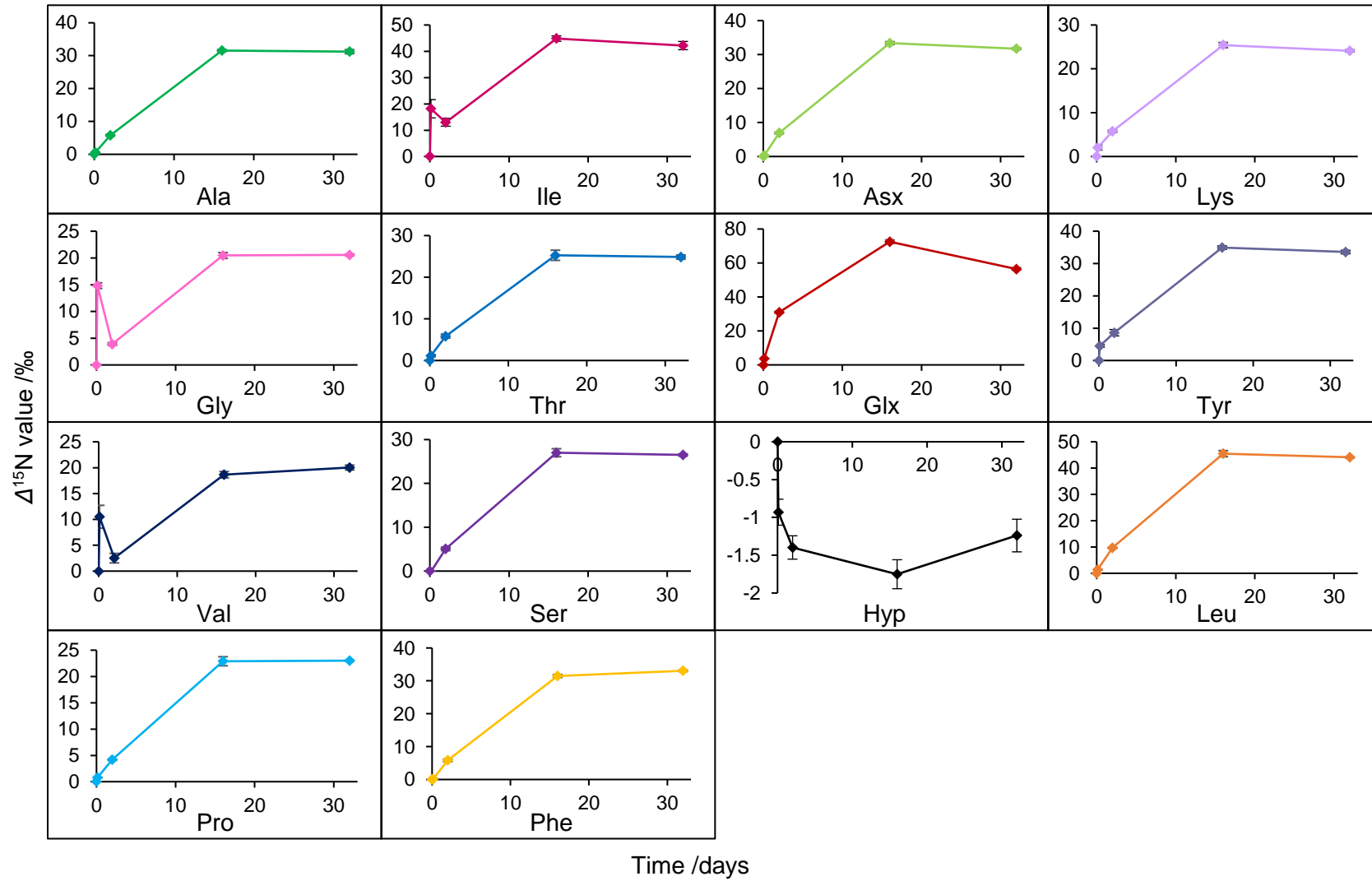

f

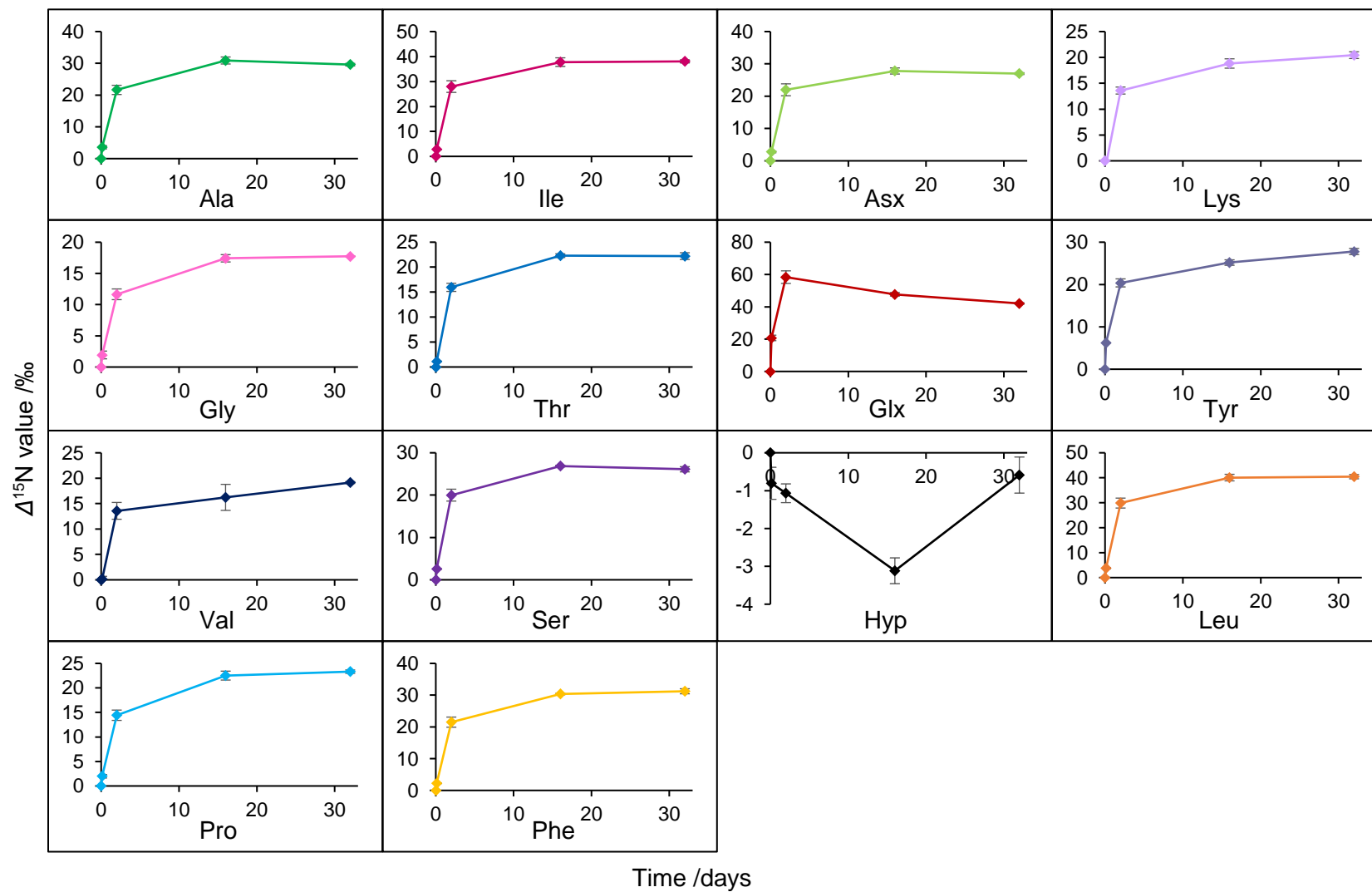

Supplement: fnae041_Supplemental_Files [file fnae041_supplemental_files.zip › S.Fig.3.pdf]
